# *Allium ursinum* L. from the apennine flora: a phytoalimurgic resource for modulating gut inflammation

**DOI:** 10.1039/d6ra04402f

**Published:** 2026-08-28

**Authors:** Simonetta Cristina Di Simone, Mariachiara Gabriele, Alessandra Acquaviva, Fatma Tunali, Annalisa Chiavaroli, Giustino Orlando, Luigi Brunetti, Lucia Recinella, Sheila Leone, Leonardo de Assunção Pinto, Thomas Gaslonde, Leandro Machado Rocha, Raimundo Gonçalves de Oliveira-Júnior, Mattia Spano, Mehmet Veysi Cetiz, Abdullahi Ibrahim Uba, Gokhan Zengin, Luigi Menghini, Claudio Ferrante

**Affiliations:** a Botanical Garden “Giardino Dei Semplici”, Department of Pharmacy, “G. D’Annunzio” Chieti-Pescara Via Dei Vestini 31 66100 Chieti Italy claudio.ferrante@unich.it simonetta.disimone@unich.it mariachiara.gabriele@phd.unich.it alessandra.acquaviva@unich.it annalisa.chiavaroli@unich.it giustino.orlando@unich.it luigi.brunetti@unich.it lucia.recinella@unich.it sheila.leone@unich.it turali.fatma@gmail.com; b Post-Graduation Program in Vegetal Biotechnology and Bioprocesses, Rio de Janeiro Federal University Rio de Janeiro CEP 21941-590 Brazil leodeasspinto@gmail.com; c Laboratory of Natural Products Technology, Department of Pharmaceutical Technology, Faculty of Pharmacy, Fluminense Federal University Niterói CEP 24241-000 Brazil leandromr@id.uff.br; d Cibles Therapeutiques et Conception de Medicaments (CiTCoM), UMR CNRS 8038, Faculty of Pharmacie, Université Paris Cité 75006 Paris France raimundo.goncalves-de-oliveira-junior@u-paris.fr thomas.gaslonde@u-paris.fr; e Department for the Promotion of Human Science and Quality of Life, San Raffaele University Via di Val Cannuta 247 Rome 00166 Italy mattia.spano@uniroma5.it; f Department of Medical Biochemistry, Faculty of Medicine, Harran University Sanliurfa 63290 Turkey mvcetiz@gmail.com; g Department of Biostatistics and Medical Informatics, Faculty of Medicine, Istinye University 34396 Istanbul Türkiye abdullahi.iu2@gmail.com; h Department of Biology, Science Faculty, Selcuk University 42130 Konya Turkey gokhanzengin@selcuk.edu.tr

## Abstract

*Allium ursinum* L. is a perennial herbaceous plant widely distributed across Europe and Asia. Its leaves are traditionally consumed as food and used in folk medicine, particularly for gastrointestinal well-being. Despite its phytochemical and ethnopharmacological similarity to *Allium sativum*, whose gastrointestinal protective effects are well documented, antioxidant and anti-inflammatory properties of *A. ursinum* in the intestinal tract remain poorly explored. This study investigated antioxidant, enzyme inhibitory, and anti-inflammatory effects of leaf polar extracts of *A. ursinum*, providing experimental support for its traditional use in food and pharmaceutical applications. Ultrasound-assisted extraction was performed using water and hydroalcoholic solvents. Extracts were characterized using chromatographic, spectroscopic, and NMR techniques. Antioxidant activity was evaluated through DPPH, ABTS, CUPRAC, and FRAP assays, while enzyme inhibition against α-glucosidase, α-amylase, tyrosinase, and cholinesterases was assessed by colorimetric methods. Anti-inflammatory effects were investigated using isolated mouse colon tissue exposed to *Escherichia coli* lipopolysaccharide (LPS), evaluating mRNA expression of IL-6, COX-2, and iNOS. The extracts displayed a complex metabolite profile dominated by sulfur-containing compounds, mainly methiin and alliin. The water extracts were rich in sugars, amino acids, and organic acids, whereas hydroalcoholic extracts preferentially concentrated polyphenols. The latter showed the strongest antioxidant and enzyme inhibitory activities, correlating with total phenolic and flavonoid content. Notably, the 50% hydroalcoholic extract significantly downregulated LPS-induced IL-6, COX-2, and iNOS gene expression in isolated colon tissue ex vivo, demonstrating anti-inflammatory activity. Overall, these findings support the use of *A. ursinum* extracts for gastrointestinal health. Further studies are warranted to define pharmacological and toxicological profile.

## Abstract 2

Leaf extracts of *Allium ursinum* may help sustain its traditional applications as a food source and in maintaining colon health.

## Graphical abstract

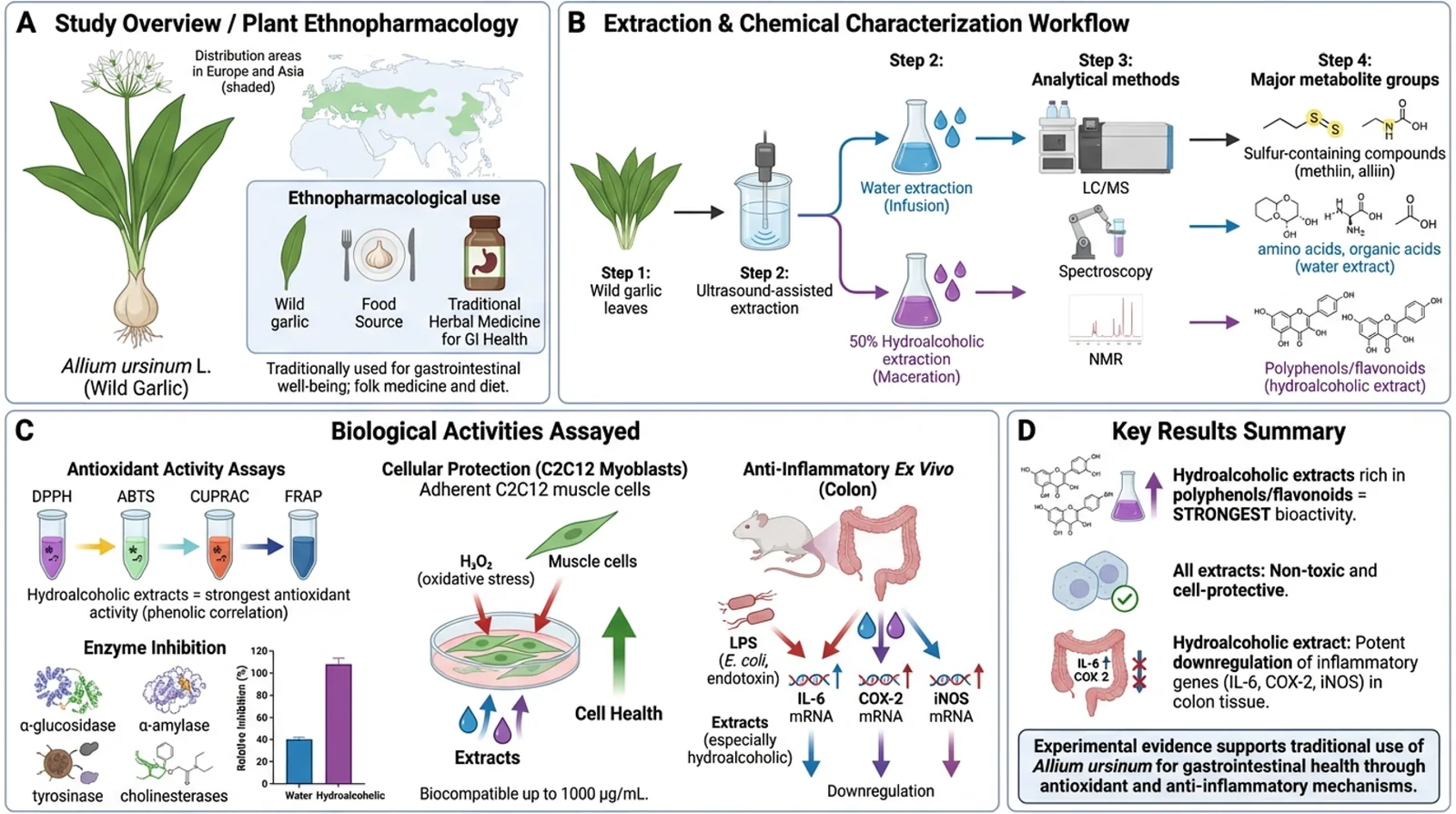

## 1 Introduction

*Allium ursinum*, wild garlic or bear garlic, is a perennial herb in the Amaryllidaceae family found across Europe and Asia. The name comes from an old story that bears ate it after hibernation to clean out their system.^1^ There are two subspecies, mostly distinguished by their pedicels: ssp. *ursinum* has rough, papillose ones and grows in western and central Europe, ssp. *ucrainicum* has smooth ones and turns up further east and southeast. It grows from February to June.^2^ The bulb lengthens to 4–6 cm, the three-sided stem reaches about 40 cm, and two or three elliptical leaves wrap around it, with white flower clusters showing up from early April to late May.^3^ It likes fertile soil in forests and mountains, usually near water,^4^ and unlike garlic it reproduces mostly by seed rather than by splitting bulbs.^1^ It can be cultivated too, and does well on chernozem soil in particular.^3^

The leaves are what people actually eat, usually young and raw in salads,^5^ and they also carry most of the plant's phenolics (kaempferol derivatives especially) and sulfur compounds.^4,6,7^ These are behind the anti-inflammatory, antioxidant, and antimicrobial effects reported in various studies.^8^ Traditional use, though, covers the whole plant, for gut, heart, and metabolic issues including diabetes and obesity.^4^ According to the Decree of the Italian Ministry of Health of August 10, 2018, the Ministry authorized bulbs, flowers, and leaves (raw or as extracts) for food supplements covering gut, cardiovascular, and respiratory support, lipid regulation, and oxidative stress. Garlic's protective effects on the gut are well documented.^8^ Wild garlic's aren't, not in any direct sense. What evidence exists is ethnopharmacological and leans on its chemical resemblance to garlic rather than actual studies in this system.^6,9^

**Fig. 1:**
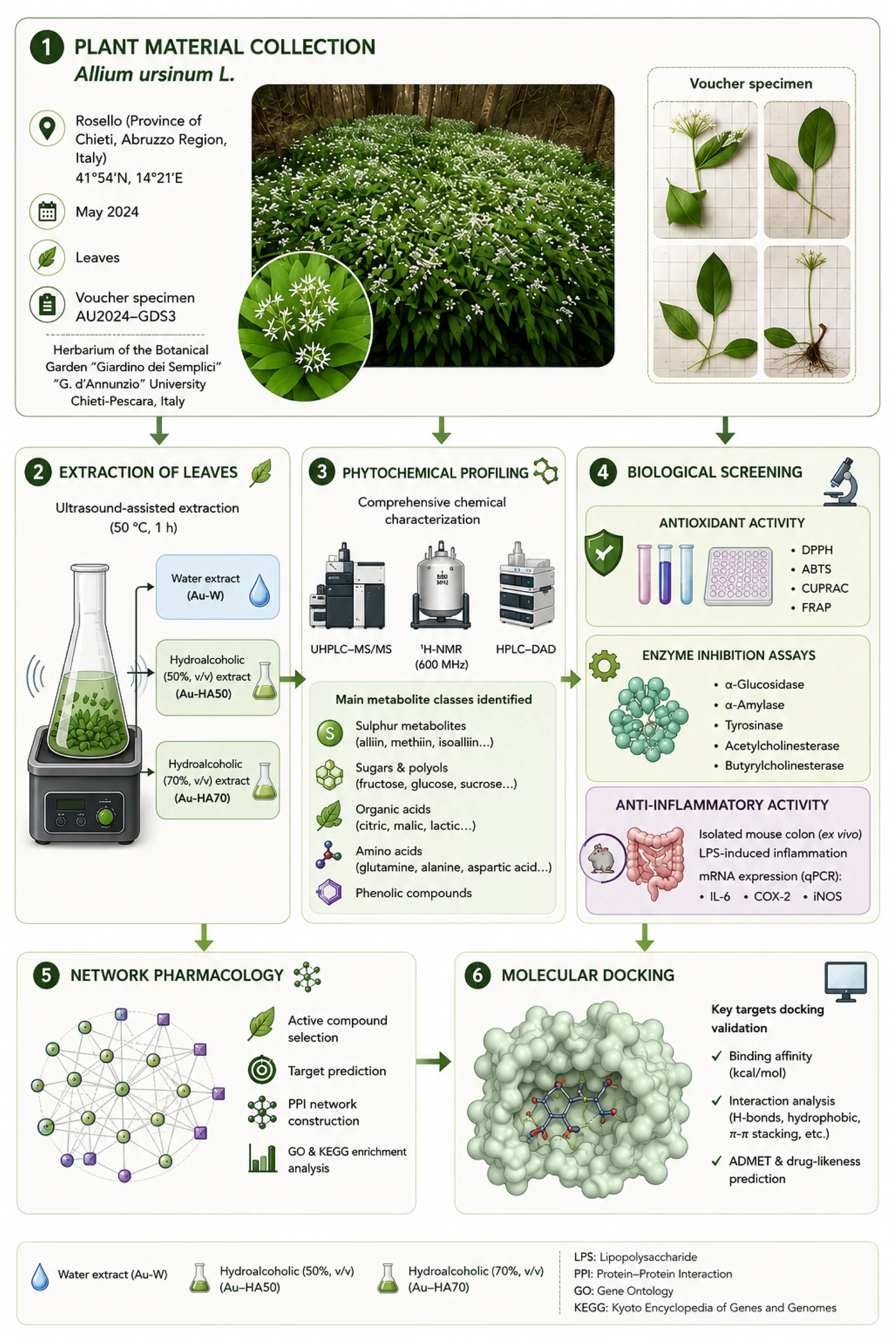
Flowchart of the study.

Therefore, the aim of this study was to explore the antioxidant, enzyme inhibitory, and anti-inflammatory effects of polar extracts of *A. ursinum* leaves, considering their traditional use in the food and pharmaceutical fields. The extracts were prepared *via* ultrasound-assisted extraction in water and hydroalcoholic solutions (50–70%, v/v), in order to mimic infusion and maceration processes, respectively. On the one hand, this choice was made to preserve the traditional health-promoting approach to the use of phytoalimurgic plants.^10^ On the other hand, the use of water and hydroalcoholic solutions as solvents was chosen to fully comply with the extraction methods reported in the pharmacopoeia. The phytochemical profile of the extracts was extensively analyzed using different and complementary analytical methods, including chromatographic and spectroscopic techniques.^11^ Scavenging/reducing and enzyme (glucosidase, amylase, tyrosinase, and cholinesterases) inhibition effects were assayed *via* colorimetric methods.^12^ Whilst anti-inflammatory effects in the colon were determined through the use of a validated *ex vivo* paradigm based on isolated colon tissue exposed to *Escherichia coli* lipopolysaccharide.^13^ In this context, the gene expression of pro-inflammatory biomarkers including interleukin-6 (IL-6), cyclooxygenase-2 (COX-2, also known as PGTS2), and inducible nitric oxide synthase (iNOS, also known as NOS2) was assessed. Finally, an *in silico* approach including target component analysis and molecular docking was performed with the aim of unraveling the mechanisms underpinning the identified biological effects. For convenience, a synthetic flowchart about the finality of the study is reported in Fig. 1.

## 2 Materials and methods

### 2.1. Plant material

**Fig. 2:**
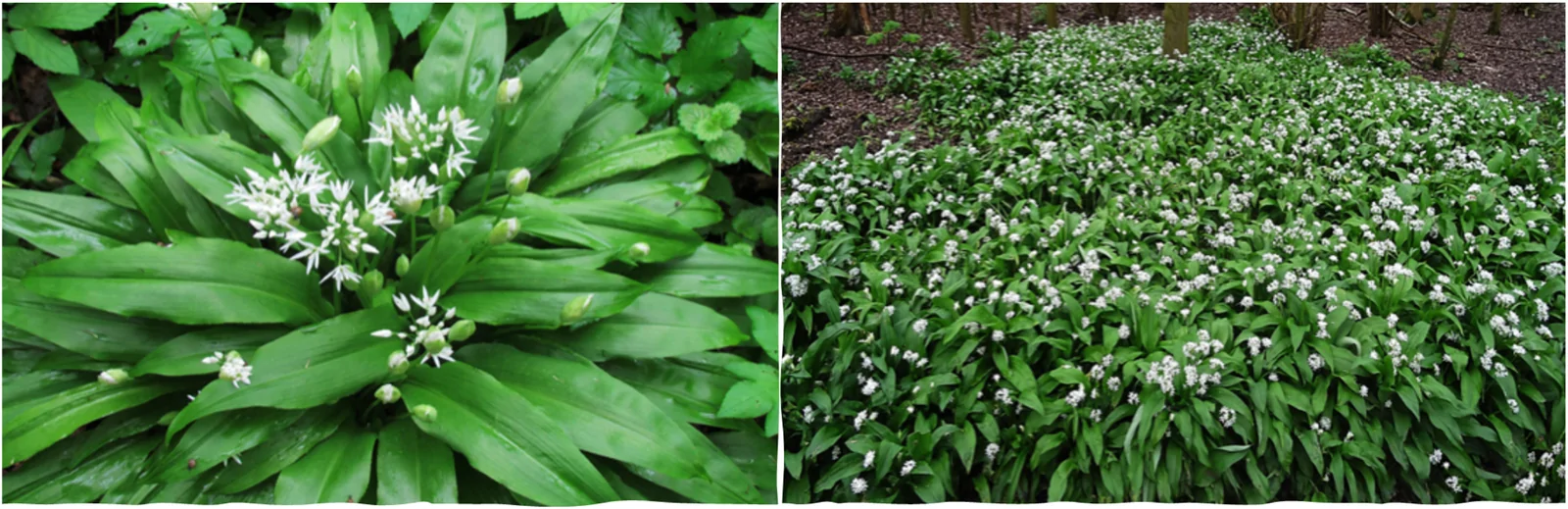
*Allium ursinum* plant population located in the woods near the village of Rosello (Province of Chieti, Abruzzo Region, Italy; 41°54′N, 14°21′E), from which the specimens used in this study were collected in May 2024.

**Fig. 3:**
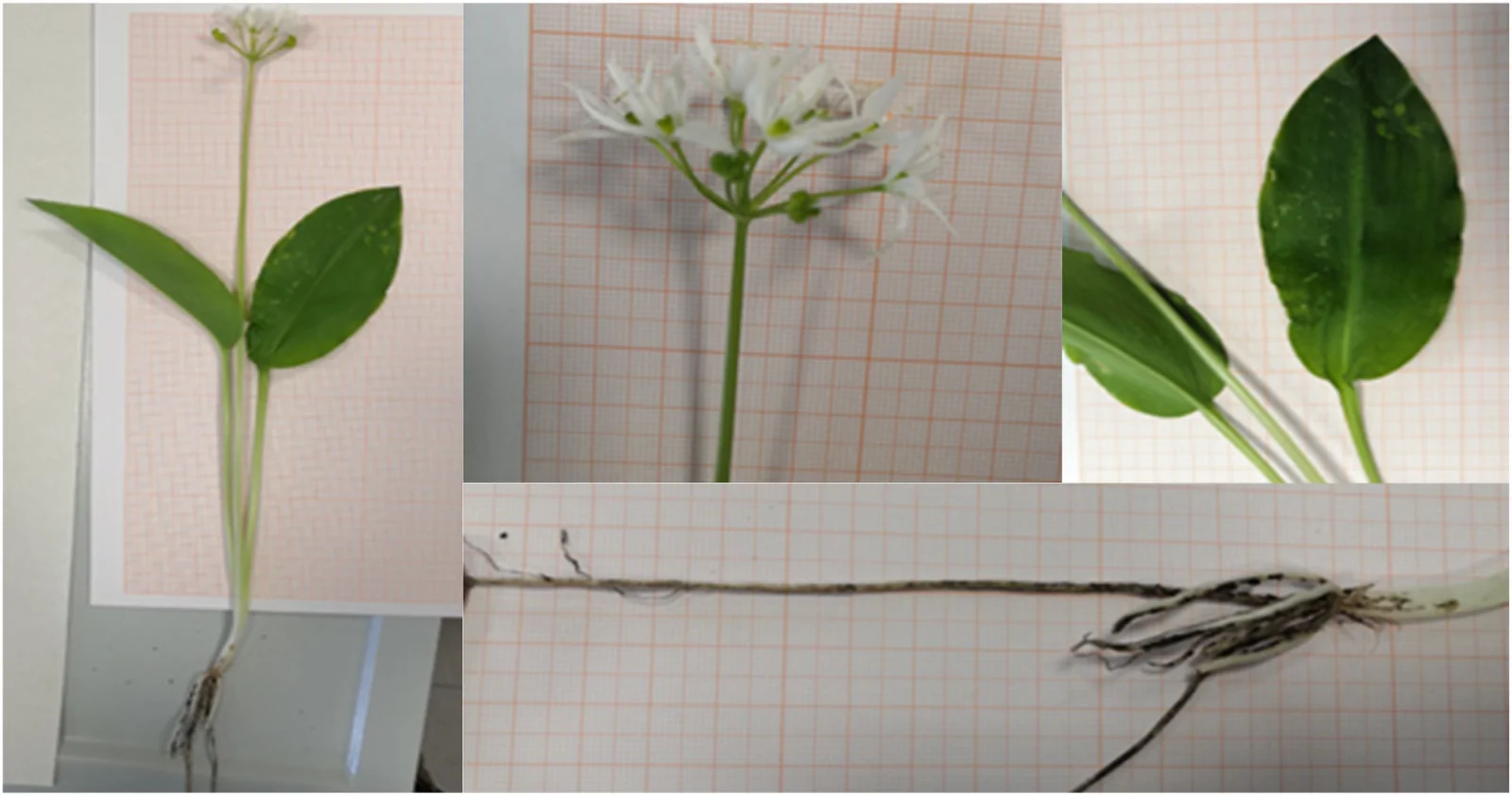
A representative sample of wild garlic (*Allium ursinum*) had the following characteristics: six flowers arranged in an umbel; stem length: 14 mm; main stem length: 25 cm (from the base of the umbel to the point where it joins the other stems); leaf stem length: 11 cm; maximum leaf width: 5.1 cm; and length of the longest root: 13.7 cm. Voucher specimen number AU2024-GDS3 deposited at the Herbarium of the Botanical Garden “Giardino dei Semplici” operated by the “G. d'Annunzio” University of Chieti-Pescara (Italy).

*Allium ursinum* L. (Amaryllidaceae) was collected in May 2024 in the area surrounding Rosello (Province of Chieti, Abruzzo Region, Italy; 41°54′N 14°21′E; approximately 920 m a.s.l.) (Fig. 2). The material was subsequently dried in a well-ventilated environment, protected from direct sunlight, for a minimum of three weeks. The selection of the harvesting site and the taxonomic identification were performed by Prof. Luigi Menghini, Prof. Claudio Ferrante, and Dr. Simonetta Cristina Di Simone. A representative voucher specimen was recovered during collection (Fig. 3) and is deposited in the Herbarium of the Botanical Garden “Giardino dei Semplici” operated by “G. d’Annunzio” University Chieti-Pescara (Italy). This herbarium is currently not registered in the *Index Herbariorum* and therefore has no official herbarium acronym. The voucher specimen is catalogued under the accession number AU2024-GDS3.

Ultrasound-assisted extraction with aqueous (70 °C for 1 h), hydroalcoholic (50%, v/v) and (70%, v/v) solutions at 50 °C for 1 h was carried out, in analogy with a previous study of ours (Menghini *et al.*, 2018). Dried plant material was extracted at a drug-to-solvent ratio of 1 : 25 (w/v), corresponding to 40 mg of dried plant material per mL of extraction solvent. The extraction yield was determined gravimetrically and expressed as the percentage of dried extract relative to the initial mass of dried plant material.

### 2.2. Chemicals

Potassium hosphate monobasic (KH_2_PO_4_), potassium phosphate dibasic (K_2_HPO_4_) and 3-(trimethylsilyl)-propionic-2,2,3,3-*d*_4_ acid sodium salt (TSP) were supplied by Merck Life Science S.r.l. (Milan, Italy). Deuterated water (D_2_O) 99.97 atom% of deuterium and deuterated methanol (CD_3_OD) 99.80 atom% of deuterium were purchased from Euriso-Top (Saclay, France).

### 2.3. NMR analysis of *Allium ursinum* extracts

One mL of Au–W extract was dried under nitrogen flow and dissolved in 1 mL D_2_O containing PBS 100 mM (pH 7.4) and TSP 1 mM as internal standard. One mL of Au-HA50 was dried under nitrogen flow and dissolved in 1 mL D_2_O/CD_3_OD 1 : 1 v/v containing TSP 1 mM as internal standard. One mL of Au-HA70 was dried under nitrogen flow and dissolved in 1 mL D_2_O/CD_3_OD 3 : 7 v/v containing TSP 1 mM as internal standard. For each solution, 700 µL were transferred to a 5 mm NMR tube for analysis. NMR analyses were carried out using a JEOL JNM-ECZ 600R spectrometer equipped with a 5 mm FG/RO DIGITAL AUTOTUNE probe. ^1^H mono-dimensional experiments were carried out using 128 scans, residual water signal presaturation, 7.7 s relaxation delay, 8.3 µs 90° pulse, 64 K data points, and 9000 Hz spectral width. The obtained spectra were processed using JEOL Delta software (v5.3.1). For quantitative analysis, integrals of the selected resonances in ^1^H NMR spectra were measured with respect to TSP integral. Results were expressed as mg g^−1^ of dried *Alium ursinum* ± SD (*n* = 3).

### 2.4. Colorimetric assays for total phenolics, total flavonoids, antioxidant effects and enzyme inhibition

The total phenolic content (TPC), total flavonoid content (TFC), and antioxidant activity [ferric reducing antioxidant power (FRAP), CUPric reducing antioxidant capacity (CUPRAC), 1,1-diphenyl-2-picrylhydrazil (DPPH), and 2,2′-azinobis-(3-ethylbenzothiazoline-6-sulfonic acid) (ABTS), phosphomolybdenum (PBD), metal chelation activity (MCA)] of the extracts were determined in accordance with previously validated analytical protocols.^14^ Enzyme inhibition assays targeting acetylcholinesterase (AChE), butyrylcholinesterase (BChE), tyrosinase, amylase, and glucosidase were performed using the tested extracts, following validated procedures described in the scientific literature. Details are reported in the SI.

### 2.5. HPLC-UV quantification of phenolic compounds

The phenolic content of the extracts was also quantified by reversed-phase HPLC-UV under gradient-elution conditions. Analyses were conducted on a Jasco chromatographic system comprising PU-2080 PLUS pumps, a DG-2080-54 degasser, a MIX-2080-32 mixer, a UV detector, an AS-2057 PLUS autosampler, and a CO-2060 PLUS column oven (Jasco, Tokyo, Japan). ChromNAV2 software was used for data acquisition and peak integration. Chromatographic separation was achieved using an Infinity Lab Poroshell 120-SB C18 column (150 × 4.6 mm i.d., 2.7 µm; Agilent, Santa Clara, CA, USA) maintained at 30 °C. The 60 min gradient run was initiated with 97% water + 0.1% formic acid and 3% methanol + 0.1% formic acid (Table 1). UV detection was performed at 254 nm. The injection volume was 5 µL. Identification was carried out through comparison with the retention time of the analytical standard. Quantification was performed using 7-point external calibration curves exhibiting excellent linearity (*R*^2^ > 0.999) in the 2–140 µg mL^−1^ range. Prior to injection, samples were diluted to a final concentration of 40 mg mL^−1^.

**Table 1:** Gradient-elution conditions applied for the HPLC-UV analysis of phenolic compounds. The UV detector wavelength was set to 254 nm

Time (min)	Composition A% (water + formic acid 0.1%)	Composition B% (methanol + formic acid 0.1%)	Flow (mL min^−1^)
1	97	3	0.6
5	77	23	0.6
12	73	27	0.6
18	57	43	0.6
25	52	48	0.6
32	50	50	0.6
34	50	50	0.6
37	35	65	0.6
40	5	95	0.6
47	10	90	0.6
48	10	90	0.6

#### 2.5.1. LC-HRMS/MS dereplication and molecular network of *Allium ursinum* hydroalcoholic (50%, v/v) extract

The hydroalcoholic (50%, v/v) extract was dissolved in HPLC-grade methanol at 10 mg mL^−1^ (w/v), sonicated for 15 min, and filtered through a 0.22 µm PTFE membrane. The injection volume was 2 µL. Chromatographic separations were carried out on a Thermo Scientific Vanquish HPLC equipped with a diode-array detector with a Zorbax Eclipse Plus C18 column (Agilent, 2.1 × 100 mm, 1.8 µm) maintained at 25 °C. The mobile phase consisted 0.1% formic acid and acetonitrile, with 0.1% formic acid, delivered at 0.3 mL min^−1^. The gradient used was from 5% to 100% at 10.5 min, the latter maintained until 12.0 min, and then re-equilibrated to initial conditions for a total run time of 12.5 min. The UV detection was performed in multichannel DAD mode from 200 to 400 nm, with individual channels monitored at 200, 220, 280, 340, and 400 nm (8 nm bandwidth, 5 Hz data collection rate). The HPLC was combined with an Orbitrap Exploris 120 mass spectrometer (Thermo Scientific) equipped with a heated electrospray ionization (H-ESI). Source parameters were as follows: ion transfer tube temperature of 325 °C, vaporizer temperature of 300 °C, and spray voltages of +3.5 kV (positive mode). Full-scan MS data were acquired at 60 000 over an *m*/*z* range of 100–1000. MS/MS acquisition was implemented for precursors exceeding 1.0 × 10^5^ and isolated with a 1.2 *m*/*z* window. They were fragmented by stepped higher-energy collisional dissociation (HCD), and the spectra were acquired at 30 000. Data procedures were performed in MZmine (4.5.3.7), and Feature-Based Molecular Networking was conducted on the GNPS and Sirius platforms. A dereplication strategy was applied, in which compound annotation was achieved through comparison of MS/MS spectra with public spectral libraries, with putative identifications. The parameters for GNPS were configured to a minimum cosine score of 0.7, however, annotation confidence was not based solely on cosine score but also supported by additional criteria, as the precursor and fragment ion mass tolerances of 0.02 Da and demand of at least six matched fragment ions and peaks for library searching. The GNPS data is available for the positive and negative ionizations, respectively, in: https://gnps.ucsd.edu/ProteoSAFe/status.jsp?task=a51184218b96460e83783647f166c9b2 and https://gnps.ucsd.edu/ProteoSAFe/status.jsp?task=52817253a347446ba9cadba8ac791056. The SIRIUS (version 6.3.3) was configured to determine molecular formula and structure annotation with predominant present elements (H, C, N, O, P, B, S, Cl, Se, Br); 10 candidates per ionization; MS/MS mass deviation of 10 ppm; *de novo* search below *m*/*z* 800, and fallback diverse adducts. Formulas were searched across databases like METACYC, COCONUT, and PUBCHEM using CSI: FingerID/COSMIC, and classes predicted by CANOPUS. The resulting molecular networks were explored in Cytoscape, where node size was mapped to precursor intensity and node color to sample superclass.

### 2.6. Allelopathy assay

The potential phytotoxic properties of *A. ursinum* extracts were assessed through an allelopathy assay. The extracts were evaluated across a concentration range of 2.5–40 mg mL^−1^. Commercial seeds of *Cichorium intybus* L. and *Dichondra repens* were selected as test species. A comprehensive description of the experimental protocol is provided in the SI.

### 2.7. Brine shrimp lethality assay

The brine shrimp (*Artemia salina*) lethality assay (BSLA) was carried out following established literature protocols.^15,16^ In brief, groups of nauplii (*n* = 10) were exposed, in triplicate, to extract concentrations ranging from 0.625 to 20 mg mL^−1^ in 5 mL of artificial seawater. After 24 h, surviving organisms were enumerated, and LC_50_ values were determined using GraphPad software (version 5.01). Control mortality rates ≤10% were considered acceptable.

### 2.8. *Daphnia magna* heart beat rate assay

In *Daphnia magna* Heart Beat Rate Assay, the extract concentration tested corresponded to the LC_50_ value determined using the Brine Shrimp Lethality Assay (BSLA). Under basal conditions, *D. magna* were exposed to the extract for 15 minutes, after which heart rates were recorded. Cardiotoxicity was induced with a 10% ethanol (EtOH) solution. In this case, organisms were pre-treated with the extract for 15 minutes and then exposed to 10% EtOH for 2 minutes before heartbeat measurements were taken. A negative control (H_2_O) and a positive control (10% EtOH) were included. All concentrations and controls were tested in triplicate.

### 2.9. Cell culture

The biocompatibility of the *A. ursinum* aqueous and hydroalcoholic extracts was assessed in human normal colon epithelial cells (WiDr, ATCC® CCL-218™) and mouse myoblasts (C2C12, ATCC® CRL-1772™) using the MTT assay. Both cell lines were sourced from the American Type Culture Collection (ATCC, Manassas, VA, USA). Detailed cell-culture procedures are reported in the SI.

### 2.10. *Ex vivo* study

The anti-inflammatory activity of *A. ursinum* extracts was assessed *ex vivo* using isolated colon tissues from 20 adult C57BL/6 mice (3 months old, 20–25 g). In agreement with the recognized principles of “Replacement, Refinement and Reduction in Animals in Research,” colon specimens were obtained as residual material from vehicle (saline)-treated mice randomized in our previous experiments, approved by local ethical committee (“G. d'Annunzio” University, Chieti, Italy) and Italian Health Ministry (Project no. F4738.N.5QP). Colon specimens were incubated for 4 h with aqueous or hydroalcoholic *A. ursinum* extracts (200–1000 µg mL^−1^) in the presence of *Escherichia coli* lipopolysaccharide (LPS, 50 µg mL^−1^). Following treatment, mRNA expression levels of COX-2, iNOS, and IL-6 were quantified by real-time RT-PCR. Comprehensive methodological details for the *ex vivo* protocol are provided in the SI.

### 2.11. *In silico* study

#### 2.11.1. Prediction of compound-related targets

The chemical structures of the identified compounds were first obtained from the PubChem database in canonical SMILES format. These SMILES representations were then submitted to the Similarity Ensemble Approach (SEA) platform to predict potential protein targets on the basis of structural similarity. All predicted targets obtained for the studied compounds were collected and merged into a single dataset, and duplicate entries were removed prior to downstream analyses. The resulting non-redundant target set was used as the basis for subsequent disease enrichment, overlap analysis, functional annotation, and network construction.

#### 2.11.2. Identification of disease-related genes and overlapping targets

Based on the disease enrichment results, Inflammatory Bowel Disease (IBD) was selected as the representative disease for subsequent analyses. IBD-related genes were retrieved from the GeneCards database using the keyword “Inflammatory Bowel Disease”. To ensure a reliable association between proteins and the disease, genes with a relevance score ≥15 were selected as myocardial Inflammatory Bowel Disease-related targets. The collected IBD-associated genes were then compared with the predicted targets of the studied compounds, and the overlapping genes were identified through intersection analysis after removal of duplicate entries. These shared genes were considered potential therapeutic targets through which the compounds may exert their effects against IBD. To further explore the interaction relationships among the overlapping genes, a protein–protein interaction (PPI) network was constructed using the STRING database V12.0. An interaction confidence score threshold of 0.4 was applied to balance network coverage and reliability. The resulting PPI network was subsequently imported into Cytoscape V3.10.3 for visualization and further topological analysis.

#### 2.11.3. GO enrichment and KEGG pathway analysis

Functional enrichment analyses were performed to better understand the biological roles of the identified targets. Gene Ontology (GO) terms and Kyoto Encyclopedia of Genes and Genomes (KEGG) pathways were analyzed using the DAVID 6.8 platform. Terms with a *P* value < 0.05 were considered statistically significant. For presentation, the ten most enriched GO categories in biological process (BP), cellular component (CC), and molecular function (MF) according to their *P* values, along with the thirty-five most significant KEGG pathways and PathwayView, were selected according to their fold enrichment. The enrichment results were visualized using the SRplot online tool and R software V4.3.3.

#### 2.11.4. Compound-target network construction

To characterize the interaction pattern between the identified compounds and the disease-relevant targets, a compound–target network was generated using the overlapping genes shared between the predicted targets of the studied metabolites and the inflammatory bowel disease-related genes. Only compound–target pairs corresponding to the overlap set were retained for network construction, and redundant interactions were excluded. The curated interaction dataset was subsequently imported into Cytoscape v3.10.3, where the network was visualized and analyzed according to standard topological parameters. Within the constructed network, nodes denoted compounds or overlapping target genes, while edges denoted the predicted associations between them.

#### 2.11.5. Molecular docking

Molecular docking analyses were conducted to evaluate the binding potential of the major phytochemicals in the plant extract against selected therapeutic protein targets. The selection of proteins was guided by their established associations with experimentally observed enzyme-inhibitory and antimicrobial activities. Specifically, AChE, BChE, α-amylase, α-glucosidase, tyrosinase, NOS2, PTGS2, and IL6 were selected, as inhibition of these enzymes is widely recognized as a therapeutic approach for the management of neurodegenerative disorders, metabolic diseases, pigmentation-related conditions, and inflammation. Additional targets associated with cancer therapy were identified by examining gene dependency patterns obtained from the DepMap Portal. Candidate proteins were subsequently filtered based on literature evidence and the availability of high-resolution three-dimensional structures in the Protein Data Bank (Table S1). Ligand structures were initially constructed using ChemDraw and subsequently subjected to geometry optimization with Avogadro (v1.2.0). Polar hydrogen atoms were added and Gasteiger charges assigned using AutoDockTools (v4.2.6) prior to docking calculations. Docking simulations were then carried out with AutoDock Vina (v1.1.2), employing an exhaustiveness parameter of 32 to improve conformational sampling. Putative ligand-binding pockets were identified using cavity predictions from the POCASA server, and the docking grid was defined accordingly. To verify the reliability of the docking protocol, co-crystallized ligands were reintroduced into their respective native binding sites, and the resulting poses were compared with experimental conformations by calculating root-mean-square deviation (RMSD) values. Detailed protein–ligand interaction patterns, including hydrogen bonds, hydrophobic contacts, and π-stacking interactions, were subsequently characterized using the Protein–Ligand Interaction Profiler (PLIP).

### 2.12. Statistical analysis

Data are reported as mean ± SD from 3–5 independent experiments, each performed in triplicate. Statistical analyses were conducted using one-way ANOVA followed by the Newman–Keuls post hoc test (GraphPad Prism, version 5.01). Differences were considered statistically significant at *P* < 0.05. For the ex vivo experiments, sample size was estimated using the Resource Equation approach. Considering five experimental groups (*T* = 5), four animals per group were included (*N* = 20), resulting in an error degree of freedom (*E* = 15), which falls within the recommended range of 10–20.

## 3 Results and discussion

### 3.1. Metabolomic profile of alliun ursinum leaf aqueous and hydroalcoholic extracts by NMR analysis

In the present study, polar extracts, specifically water and hydroalcoholic preparations, were obtained from *Allium ursinum* leaves using an ultrasound-assisted extraction method and subsequently characterized from both phytochemical and biological perspectives. The extraction yields were 35.05 ± 0.75%, 27.90 ± 0.53%, and 36.77 ± 0.91% for the aqueous, 50% hydroalcoholic, and 70% hydroalcoholic extracts, respectively (mean ± SD, *n* = 3). The phytochemical composition of the extracts was comprehensively investigated through a set of complementary analytical approaches, including chromatographic and spectroscopic techniques.^11^

^1^H spectral information (signal chemical shifts, multiplicity, *J* coupling constants) and literature data regarding NMR analysis of garlic^11,17^ allowed to identify metabolites belonging to several classes. In particular, five sulfur metabolites (alliin, cycloalliin, isoalliin, methiin, propiin), five sugars (fructose, galactose, glucose, mannitol, sucrose), seven organic acids (acetic, citric, formic, fumaric, lactic, malic, pyruvic), twelve amino acids (alanine, asparagine, glutamate, glutamine, histidine, isoleucine, leucine, phenylalanine, threonine, tryptophan, tyrosine, valine), and other metabolites (betaine, choline, GABA, polyphenols) were identified and quantified (Table 2). NMR analysis allowed to identify a rich metabolite profile in *A*. *ursinum* extracts, with both qualitative and quantitative differences depending on extraction approach. Sulfur compounds are the most typical class of molecules of this matrix, since they are responsible for the particular organoleptic properties of the matrix as well as for several potential biological activities.^18^ Methiin and alliin were confirmed as the main sulfur compounds of the species, as previously reported,^19^ with a significative content increase and decrease with hydroalcoholic extractions for alliin and methiin, respectively. Cycloalliin, isoalliin, and propiin were measured in water extract but not in the hydroalcoholic ones. Signals typical of phenol compounds were detected exclusively in the hydroalcoholic extracts. Nevertheless, due to signal overlaps and relative low concentration, it was not possible to carry out a specific identification and quantification of the compounds in the ^1^H NMR spectra Consequently, only the main signal of these compounds, identified as unknown phenols (UP), were reported in Table 2. Among sugars and polyols, an interesting result was obtained comparing water and hydroalcoholic extracts. Galactose, glucose, fructose, and mannitol were measured only in water extract, as expected by their higher solubility in this solvent. Anyway, in the case of sucrose, a significant increase in its content was measured in both 50% v/v and 70% v/v hydroalcoholic extracts. This increase was probably due to structural and enzymatic alterations induced by the alcoholic fraction, which allowed the extraction of higher concentrations of disaccharides. Finally, for amino acids, organic acids, betaine, choline, and GABA, higher amounts were obtained, as expected, in the water extract.

**Table 2:** Metabolites identified in 600.17 MHz ^1^H-NMR spectra of water and hydroalcoholic extracts of *Allium ursinum* leaves. Quantitative results are expressed as mg g^−1^ of dried leaves ± SD (*n* = 3)

Metabolite	^1^H ppm, multiplicity, *J* [Hz]	Water extract	Hydroalcoholic (50%, v/v) extract	Hydroalcoholic (70%, v/v) extract
**Sulphur metabolites**				
Alliin	5.52, dq [1.2; 17.8]	5.49 ± 0.02	8.14 ± 0.12	8.64 ± 0.16
Cycloalliin	1.42, d [6.5]	1.07 ± 0.01	—	—
Isoalliin	6.55, dq [15.2, 1.6]	1.96 ± 0.03	1.71 ± 0.02	1.68 ± 0.03
Methiin	s, 2.84	8.51 ± 0.20	2.49 ± 0.06	1.83 ± 0.05
Propiin	1.09, t [7.4]	0.23 ± 0.01	—	—

**Sugars and polyols**				
Fructose	4.11, m	5.16 ± 0.22	—	—
Galactose	4.60, d [8.0]	0.40 ± 0.01	—	—
Glucose	4.66, d [8.0]	19.07 ± 0.05	—	—
Mannitol	3.90, m	17.31 ± 0.36	—	—
Sucrose	5.24, dd [3.9]	3.26 ± 0.02	40.27 ± 0.82	45.05 ± 1.03

**Organic acids**				
Acetic	1.92, s	0.47 ± 0.01	0.36 ± 0.02	0.16 ± 0.01
Citric	2.56, d [16.0]	9.23 ± 0.04	6.02 ± 0.3	1.59 ± 0.01
Fumaric	6.53, s	0.09 ± 0.01	0.13 ± 0.01	—
Formic	8.46, s	0.06 ± 0.01	0.12 ± 0.01	0.37 ± 0.01
Lactic	1.33, d [6.6]	0.94 ± 0.02	—	—
Malic	4.30, dd [10.0; 3.1]	4.50 ± 0.13	3.10 ± 0.08	2.38 ± 0.04
Pyruvic	2.41, s	0.29 ± 0.01	—	—

**Amino acids**				
Alanine	1.48, d [7.2]	2.96 ± 0.04	1.29 ± 0.02	1.75 ± 0.04
Asparagine	2.95, dd [16.9; 7.4]	1.18 ± 0.01	—	—
Glutamate	2.36, m	3.89 ± 0.02	—	—
Glutamine	2.46, m	12.80 ± 0.25	9.35 ± 0.32	8.04 ± 0.08
Histidine	7.11, 2	1.10 ± 0.01	—	—
Isoleucine	1.02, d [7.1]	1.48 ± 0.04	0.76 ± 0.01	0.58 ± 0.02
Leucine	0.96, d [6.2]	3.33 ± 0.02	0.78 ± 0.02	0.51 ± 0.01
Phenylalanine	7.43, m	3.15 ± 0.11	1.27 ± 0.03	1.07 ± 0.02
Threonine	1.33, d [6.6]	1.64 ± 0.07	0.52 ± 0.01	0.42 ± 0.02
Tryptophan	7.55, d [8.1]	1.14 ± 0.01	0.69 ± 0.02	0.92 ± 0.01
Tyrosine	6.90, d [8.6]	1.28 ± 0.03	0.55 ± 0.01	0.15 ± 0.01
Valine	1.05, d [7.1]	2.12 ± 0.04	0.86 ± 0.01	0.78 ± 0.01

**Other metabolites**				
Betaine	3.27, s	0.41 ± 0.01	—	—
Choline	3.21, s	0.95 ± 0.03	0.45 ± 0.01	0.46 ± 0.02
GABA	2.30, t [7.4]	1.42 ± 0.02	0.87 ± 0.03	0.70 ± 0.02
UP 1^a^	7.93, d [8.3]	—	A	A
UP 2	8.01, d [8.8]	—	A	A
UP 3	8.05, d [8.9]	—	A	A
UP 4	8.06, d [8.9]	—	A	A

a UP: Unknown phenol; A: assigned in the spectrum but not quantified.

### 3.2. Quantitative analysis of phenolic compounds and evaluation of antioxidant and enzyme inhibitory activities in water and hydroalcoholic extracts of *Allium ursinum* leaves

**Fig. 4:**
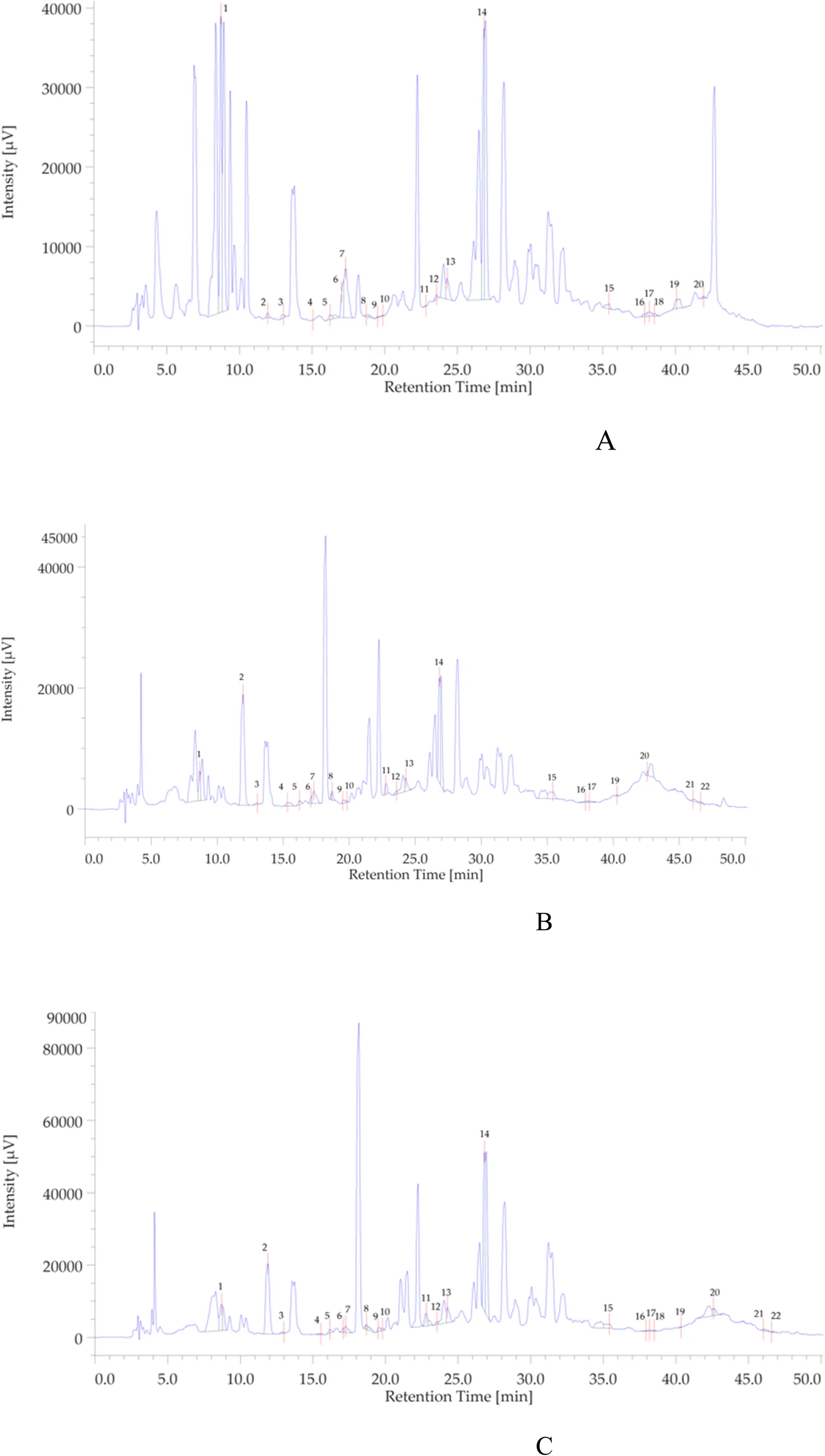
Chromatographic analysis of 22 diverse phenolic compounds identified and quantified in water (A) hydroalcoholic (50%, v/v) (B), and hydroalcoholic (70%, v/v) (C) extract from *Allium ursinum* leaves.

Water and hydroalcoholic (50%, 70%, v/v) extracts were also assayed for their total content in phenols and flavonoids, through colorimetric methods. The results reported in Table 3 showed the highest level of these compounds in the hydroalcoholic (70%, v/v) extract, gradually followed by hydroalcoholic (50%, v/v) and water extracts. The highest extraction efficacy of the hydroalcoholic solvent could be a result likely attributable to its relatively lower polarity, which enhances the solubilization and subsequent release of phenolic constituents from the matrix. However, the accuracy of total phenol quantification using only colorimetric assays has been increasingly scrutinized, as these methods require validation using chromatographic techniques to ensure their analytical specificity. Indeed, the Folin–Ciocalteu reagent is susceptible to reduction by a wide range of matrix constituents, including peptides and other non-phenolic reducing agents, thus introducing a significant positive bias in the measured values.^20^ Additionally, also other independent physicochemical variables, including temperature and extraction methods, could influence the total phenol content in *A. ursinum* extracts.^21^ To circumvent these interferences and achieve compound-specific resolution, the phenolic profile of the extracts was quantified using HPLC-UV (Table 4; Fig. 4A–C). Chromatographic analysis of 22 structurally diverse phenolic analytes, including phenolic acids, hydroxycinnamic derivatives, and multiple flavonoid subclasses, yielded similar results, with the hydroalcoholic extract (70%, v/v) as the richest in phenolic compounds (Fig. 4D). These findings are also consistent with the ^1^HNMR data reported in Table 2, where the results of phenolic content determination are also presented. The total content in phenolics could also explain the intrinsic biological effects displayed by the extracts (Tables 5 and 6). Indeed, the hydroalcoholic (70%, v/v) extract was also the most effective as scavenging/reducing agent in different assays, namely DPPH, ABTS, CUPRAC, and FRAP where it displayed the highest trolox equivalent (TE) values. The antiradical effects displayed by the extracts strongly agree with the content in phenols and flavonoids. The extracts' bioactivity was substantiated through enzyme inhibition assays, as well. In this framework, inhibitory activities were determined against a panel of key enzymes (glucosidase, amylase, cholinesterase, and tyrosinase) that are widely recognized for their involvement in inflammatory processes and oxidative stress-related pathologies. Consistent with the antioxidant evaluations, the hydroalcoholic extracts exhibited superior inhibitory potential compared with the aqueous extract. Nevertheless, the enzyme inhibitory profiles only partially correlated with the total phenolic content and the free radical–scavenging capacities.^22^ This could be related, albeit partially, to other non-phenolic compounds present in the extracts, among which sulfur compounds.^23,24^ In this regard, it is worth emphasizing that the concentration of sulfur compounds is not directly correlated with the polarity of the solvent, unlike what is observed for polyphenols (Table 2). This consideration may further account for the reduced prominence of differences in enzymatic inhibitory properties among the various extracts, in contrast to the clearer distinctions observed in the antioxidant assays.

**Table 3:** Total phenolic (TPC) and flavonoid content (TFC) of *A. ursinum* water and hydroalcoholic extracts. Values are reported as mean ± SD of three parallel experiments. TPC: Total phenolic content; TFC: total flavonoid content; GAE: Gallic acid equivalent; RE: Rutin equivalent

Solvents	TPC (mg GAE per g dry extract)	TFC (mg RE per g dry extract)
Water extract	16.54 ± 0.49	11.87 ± 0.20
Hydroalcoholic (50%, v/v) extract	11.46 ± 0.05	12.89 ± 0.35
Hydroalcoholic (70%, v/v) extract	15.70 ± 0.25	18.21 ± 0.10

**Table 4:** Peak number (#), Retention times (*R*_t_, min), areas (µVsec) and quantities (µg mL^−1^) of 22 identified phenolic compounds in *Allium ursinum* extracts (n.q. = not quantified, n.i. = not identified). Quantites are expressed as means of two replicates ± SD

Compounds	Peak #	*R*_*t*_ (min)	Water extract							Hydroalcoholic (50%, v/v) extract							Hydroalcoholic (70%, v/v) extract						
			Area (µVsec)	Quantity (µg mL^−1^)			Quantity (mg g^−1^ DE)			Area (µVsec)	Quantity (µg mL^−1^)			Quantity (mg g^−1^ DE)			Area (µVsec)	Quantity (µg mL^−1^)			Quantity (mg g^−1^ DE)		
Gallic acid	1	8.69	347 601	21.02	±	0.17	0.53	±	0.00	45 195	2.76	±	0.02	0.07	±	0.00	96 042	5.83	±	0.31	0.15	±	0.01
3-Hydroxytyrosol	2	11.922	5259	4.94	±	1.45	0.12	±	0.04	325 553	300.59	±	1.15	7.51	±	0.03	353 763	328.04	±	0.16	8.20	±	0.00
Caftaric acid	3	13.029	6551	1.18	±	0.04	0.03	±	0.00	4410	0.94	±	0.04	0.02	±	0.00	4979	1.00	±	0.04	0.03	±	0.00
Catechin	4	15.283	72	0.06	±	0.02	0.00	±	0.00	15 753	13.22	±	0.65	0.33	±	0.02	3734	3.13	±	0.10	0.08	±	0.00
4-Hydroxybenzoic acid	5	16.204	9952	0.03	±	0.01	0.00	±	0.00	10 547	0.04	±	0.01	0.00	±	0.00	14 664	0.12	±	0.00	0.00	±	0.00
Loganic acid	6	17.054	49 253	8.53	±	0.02	0.21	±	0.00	11 150	1.92	±	0.29	0.05	±	0.01	9698	1.67	±	0.06	0.04	±	0.00
Chlorogenic acid	7	17.288	120 010	15.64	±	0.30	0.39	±	0.01	39 886	5.55	±	0.08	0.14	±	0.00	27 424	3.98	±	0.20	0.10	±	0.01
Vanillic acid	8	18.701	4750	0.01	±	0.00	0.00	±	0.00	14 011	0.35	±	0.01	0.01	±	0.00	11 765	0.27	±	0.03	0.01	±	0.00
Caffeic acid	9	19.503	n.q.							7184	0.32	±	0.00	0.01	±	0.00	14 380	0.72	±	0.02	0.02	±	0.00
Epicatechin	10	19.829	871	0.44	±	0.01	0.01	±	0.00	3445	2.36	±	0.42	0.06	±	0.01	7063	5.07	±	0.08	0.13	±	0.00
Chicoric acid	11	22.792	1037	0.16	±	0.00	0.00	±	0.00	21 697	1.53	±	0.03	0.04	±	0.00	47 062	3.21	±	0.01	0.08	±	0.00
*p*-Coumaric acid	12	23.551	5116	0.55	±	0.12	0.01	±	0.00	6569	0.76	±	0.01	0.02	±	0.00	9753	1.20	±	0.01	0.03	±	0.00
*t*-Ferulic acid	13	24.274	37 269	2.60	±	0.06	0.06	±	0.00	29 251	2.01	±	0.05	0.05	±	0.00	58 174	4.12	±	0.08	0.10	±	0.00
Benzoic acid	14	26.811	283 969	88.58	±	0.48	2.21	±	0.01	164 960	50.83	±	1.36	1.27	±	0.03	361 550	117.53	±	1.79	2.94	±	0.04
*t*-Cinnamic acid	15	35.393	12 259	0.23	±	0.02	0.01	±	0.00	36 775	0.97	±	0.03	0.02	±	0.00	24 712	0.61	±	0.35	0.02	±	0.01
Quercetin	16	37.840	5244	1.78	±	0.02	0.04	±	0.00	4060	1.74	±	0.01	0.04	±	0.00	1704	1.65	±	0.01	0.04	±	0.00
Naringenin	17	38.104	11 107	1.13	±	0.01	0.03	±	0.00	4364	0.01	±	0.01	0.00	±	0.00	4882	0.09	±	0.01	0.00	±	0.00
2,3-Dimethylbenzoic acid	18	38.517	5287	1.47	±	0.03	0.04	±	0.00	n.i.							2079	0.91	±	0.02	0.02	±	0.00
Hesperetin	19	40.192	29 085	7.39	±	0.02	0.18	±	0.00	1466	0.61	±	0.01	0.02	±	0.00	936	0.48	±	0.03	0.01	±	0.00
Kaempferol	20	42.345	2727	1.78	±	0.01	0.04	±	0.00	12 334	2.10	±	0.34	0.05	±	0.01	23 806	2.48	±	0.02	0.06	±	0.00
Flavone	21	46.008	n.i.							7295	0.07	±	0.02	0.00	±	0.00	9870	0.13	±	0.01	0.00	±	0.00
3-Hydroxyflavone	22	46.590	n.i.							3391	1.05	±	0.04	0.03	±	0.00	1300	0.96	±	0.01	0.02	±	0.00

**Table 5:** Antioxidant properties of *Allium ursinum* water and hydroalcoholic extracts. Values are reported as mean ± SD of three parallel measurements. TE: Trolox equivalent; EDTAE: EDTA equivalent; na: not active. ABTS, 2,2′-azino-bis(3-ethylbenzothiazoline) 6-sulfonic acid; CUPRAC, cupric ion reducing antioxidant capacity; DPPH, 1,1-diphenyl-2-picrylhydrazyl; FRAP, ferric ion reducing antioxidant power; MCA, metal chelating activity; PBD, phosphomolybdenum activity

Solvents	DPPH (mg TE per g dry extract)	ABTS (mg TE per g dry extract)	CUPRAC (mg TE per g dry extract)	FRAP (mg TE per g dry extract)	MCA (mg EDTAE per g dry extract)	PBD (mmol TE per g dry extract)
Water extract	9.76 ± 0.33	40.66 ± 0.18	21.65 ± 0.38	16.52 ± 0.20	13.36 ± 0.42	1.61 ± 0.04
Hydroalcoholic (50%, v/v) extract	13.94 ± 0.58	55.31 ± 1.00	31.19 ± 0.24	22.73 ± 0.14	24.96 ± 0.05	1.37 ± 0.01
Hydroalcoholic (70%, v/v) extract	20.84 ± 0.59	61.49 ± 0.44	41.07 ± 0.89	27.94 ± 0.21	21.77 ± 0.78	1.56 ± 0.07

**Table 6:** Enzyme inhibitory of *Allium ursinum* water and hydroalcoholic extracts. Values are reported as mean ± SD of three parallel measurements. GALAE: Galantamine; KAE: Kojic acid; ACAE: Acarbose equivalent. AChE: Acetylcholinesterase; BChE: Butrylcholinesterase

Solvents	AChE (mg GALAE per g extract)	BChE (mg GALAE per g extract)	Tyrosinase (mg KAE per g extract)	Amylase (mmol ACAE per g extract)	Glucosidase (mmol ACAE per g extract)
Water extract	1.70 ± 0.03	1.63 ± 0.15	29.95 ± 1.53	0.028 ± 0.002	0.22 ± 0.01
Hydroalcoholic (50%, v/v) extract	2.68 ± 0.02	1.92 ± 0.05	60.30 ± 0.41	0.184 ± 0.003	1.65 ± 0.01
Hydroalcoholic (70%, v/v) extract	2.58 ± 0.02	1.79 ± 0.12	64.28 ± 0.85	0.206 ± 0.002	0.88 ± 0.04

### 3.3. Biocompatibility evaluation

#### 3.3.1. Eco-toxicological studies

**Fig. 5:**
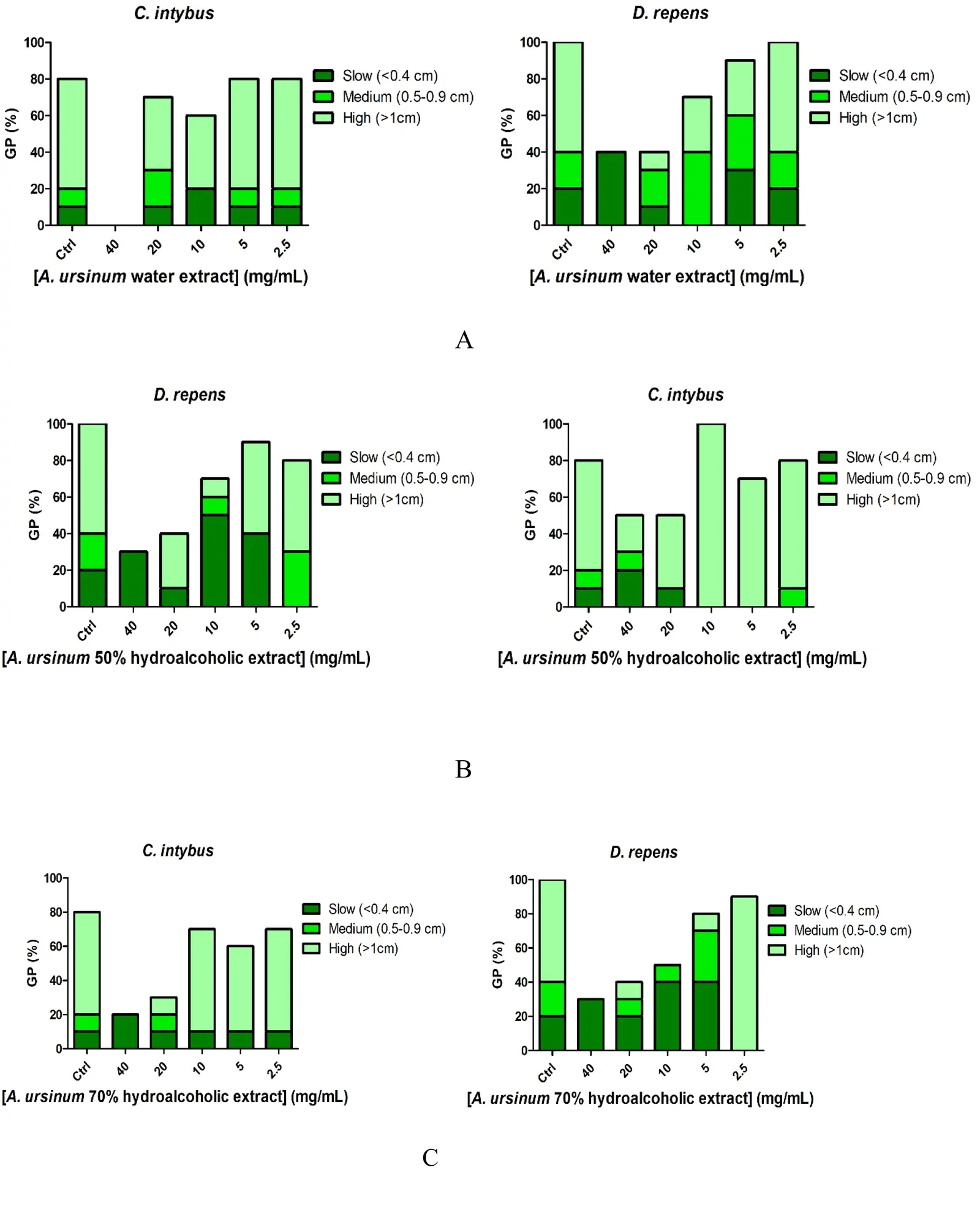
Phytotoxic effects of water (A), hydroalcoholic (50%, v/v) (B), and hydroalcoholic (70%, v/v) (C) *Allium ursinum* leaf extracts (2.5–40 mg mL^−1^) on *Cichorium intybus* and *Dichondra repens*, expressed as growth percentage (GP) inhibition, in the allelopathy assay. All extracts were biocompatible up to 10 mg mL^−1^, except for the 70% (v/v) hydroalcoholic extract on *D. repens*, which showed phytotoxicity at this concentration, with plant growth falling below 60% relative to the control (Ctrl).

**Fig. 6:**
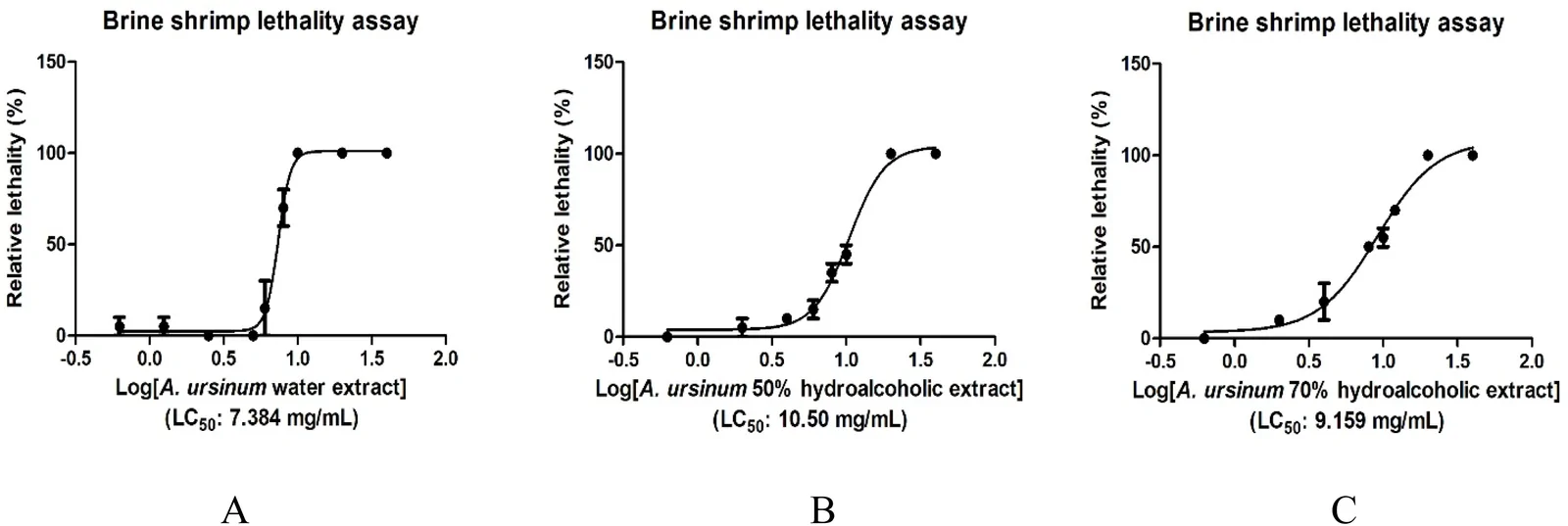
Dose–response curve displaying the lethality effects induced by water (A), hydroalcoholic (50%, v/v) (B), and hydroalcoholic (70%, v/v) (C) extracts (0.625–10 mg mL^−1^) on brine shrimps (*Artemia salina*). LC_50_ values were 7.384, 10.50, and 9.159 mg mL^−1^, respectively.

**Fig. 7:**
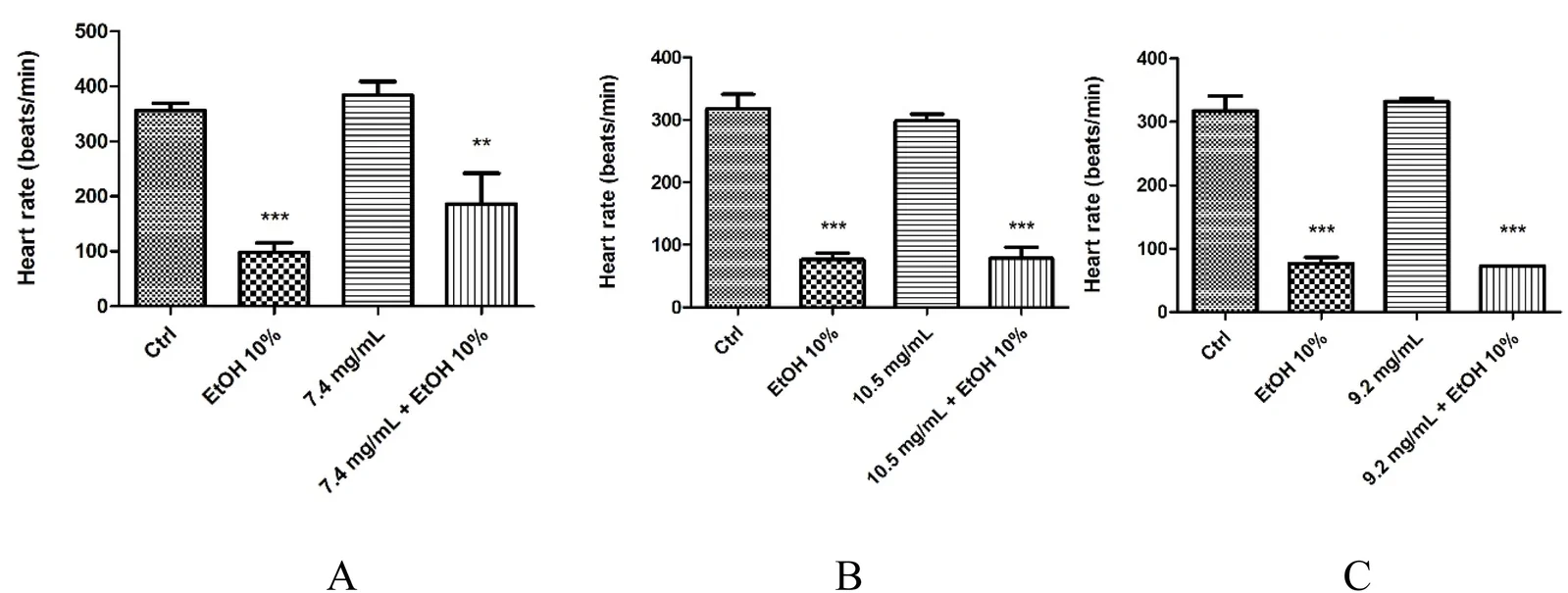
Acute exposure of *Daphnia magna* to water (A), hydroalcoholic (50%, v/v) (B), and hydroalcoholic (70%, v/v) (C) extracts at 7.384, 10.50, and 9.159 mg mL^−1^, respectively. The extracts did not induce cardiotoxic effects, in the crustaceans, although they did not prevent the ethanol-induced reduction in heart rate. Data are reported as means ± SEM. ANOVA, *P* < 0.0001. Newman–Keuls multiple comparison post hoc test ***P < 0.001 *vs.* negative Ctrl group.

To further assess the biological properties of the extracts and define their biocompatibility in eukaryotic systems, a series of ecotoxicological assays was conducted. These included allelopathic assay, brine shrimp (*Artemia salina*) lethality test, and *Daphnia magna* cardiotoxicity test. Together, these models provided complementary evidence regarding the safety and ecological impact of the preparations. In the allelopathic assay, the phytotoxic activity of the extracts (2.5–40 mg mL^−1^) was examined in two herbaceous edible species, namely *Cichorium inthybus* and *Dichondra repens*, as previously reported.^11^ Growth percentage (GP) inhibition was quantified after 96 hours of exposure. All extracts were biocompatible up to 10 mg mL^−1^, with the exception of the hydroalcoholic extract (70% v/v) tested on *D. repens* (Fig. 5A–C). In this case, phytotoxic effects became evident at 10 mg mL^−1^, and plant growth dropped below 60% at 10 mg L^−1^ when compared with the control group (Ctrl). This effect may result from the species-specific sensitivity and relatively high concentration of phenolic compounds in the extract, since phenolics are widely recognized for their allelopathic activity in plant–plant interactions.^25^ The current results also add to literature data indicating the ability of *A. ursinum* bulbs and leaves to influence the growth of other herbaceous plants through the release of volatile and phenolic compounds into the surrounding environment.^26^ The brine shrimp *A. salina* is a crustacean inhabiting saline environments and is widely used in toxicity testing. Its easy cultivation, rapid lifecycle, and high sensitivity to diverse xenobiotics, including plant extracts, make it a simple and reliable model for assessing acute toxicity.^16^ The potential toxicity of xenobiotics in the brine shrimp assay is evaluated according to the classifications proposed by Meyer and Clarkson. Meyer and colleagues^16^ distinguish non-toxic and toxic substances based on LC_50_ thresholds: compounds with LC_50_ > 1000 µg mL^−1^ are considered non-toxic, whereas those with LC_50_ < 1000 µg mL^−1^ are regarded as toxic. Clarkson and colleagues^27^ further refined this approach by defining four toxicity categories: non-toxic: LC_50_ > 1000 µg mL^−1^; low toxicity: 500 < LC_50_ < 1000 µg mL^−1^; moderate toxicity: 100 < LC_50_ < 500 µg mL^−1^; high toxicity: LC_50_ < 100 µg mL^−1^. According to these classications, all tested extracts were biocompatible, with LC_50_ values in the range 7.38–10.50 mg mL^−1^ (Fig. 6A–C, Table 7). These values were used as reference points for the subsequent assay in the *Daphnia magna* model*. Daphnia magna* are planktonic, filter-feeding crustaceans that typically inhabit standing freshwater systems, where they feed on suspended microorganisms such as bacteria, algae, cyanobacteria, and protozoans. Owing to their long history in scientific study, they are among the most established model organisms in biological research. Their pronounced sensitivity to environmental and chemical stressors, including pharmaceuticals, renders their behavioral and physiological responses robust and widely utilized biomarkers in ecotoxicological assessments.^28^ The crustaceans were exposed to the extracts at their respective LC_50_ values, either in the absence or in the presence of 10% (v/v) ethanol, which was included as a reference cardiotoxic stimulus known to reduce heart rate.^29^ The assay further confirmed the biocompatibility of *A. ursinum* extracts, as they produced no effect on heart rate, although they did not prevent the ethanol-induced reduction in heart rate (Fig. 7A–C). Overall, the results of the ecotoxicological assessments provide a more comprehensive view of the extracts' bioactivity and safety profile, with the observed differences being partly attributable to their total phenolic content. Nevertheless, the ecotoxicological models employed in this study should be regarded only as preliminary indicators of biocompatibility and are not entirely exhaustive, as tissue damage biomarkers were not evaluated. Therefore, following the approach adopted in previous studies,^11^ a concentration at least 5–10 fold lower than the LC_50_ value obtained from the brine shrimp lethality assay was selected as a putative biocompatibility threshold for the *in vitro* assays described herein.

**Table 7:** Brine shrimp lethality assay results in terms of LC_50_ value and toxicity levels according to Meyer's and Clarckson's classifications. Tested samples: *Allium ursinum* water and hydroalcoholic (50%, v/v) and (70%, v/v) extracts, in a concentration range between 0.625 and 40 mg mL^−1^. All the extracts can be classified as non-toxic against *Artemia salina* nauplii, with LC_50_ values of 7.40 mg mL^−1^,10.50 mg mL^−1^, and 9.16 mg mL^−1^, respectively

Tested extract	Concentration [mg mL^−1^]	LC_50_ (mg mL^−1^)	95% confidence interval	*R* ^2^	Toxicity class	
					Meyer's classification	Clarkson's classification
Water extract	[0.625–40]	7.40	6.94–7.85	0.98	Non-toxic	Non-toxic
Hydroalcoholic (50%, v/v) extract	[0.625–40]	10.50	6.29–7.29	0.97	Non-toxic	Non-toxic
Hydroalcoholic (70%, v/v) extract	[0.625–40]	9.16	9.36–11.77	0.98	Non-toxic	Non-toxic

#### 3.3.2. *In vitro* study

**Fig. 8:**
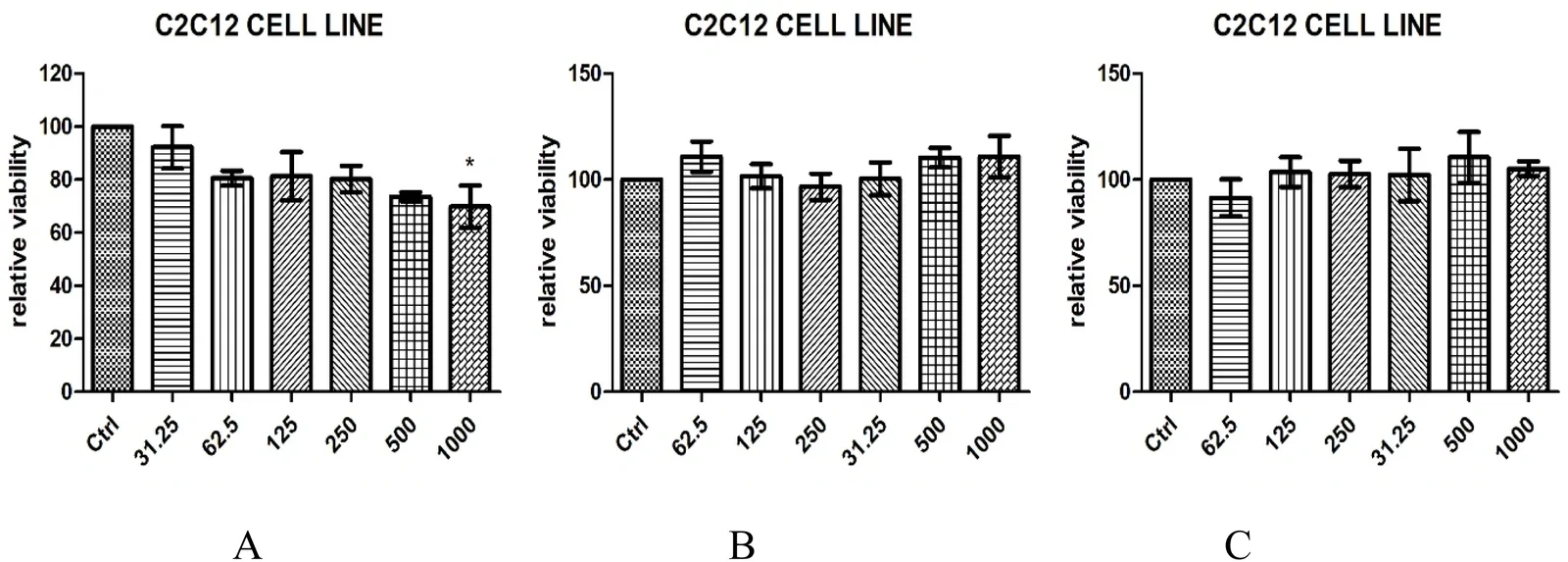
MTT assay of C2C12 cells exposed to *Allium ursinum* water extract (A), hydroalcoholic (50%, v/v) (B), and hydroalcoholic (70%, v/v) (C) extracts in range from 31.25 to 1000 µg mL^−1^, in basal conditions. Data are reported as means ± SEM. ANOVA, *P* < 0.0001; Newman–Keuls multiple comparison post hoc test, *P < 0.05 *vs.* Ctrl.

**Fig. 9:**
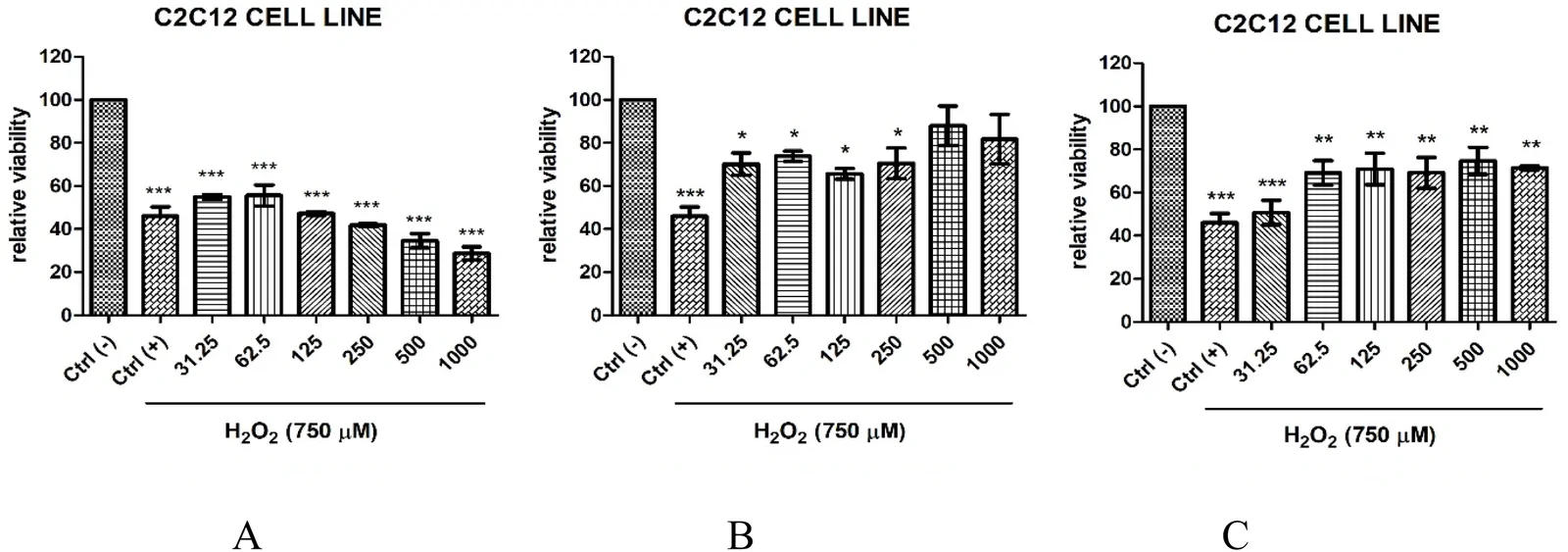
MTT assay of C2C12 cells exposed to *Allium ursinum* water extract (A), hydroalcoholic (50%, v/v) (B), and hydroalcoholic (70%, v/v) (C) extracts in range from 31.25 to 1000 µg mL^−1^, after H_2_O_2_ pre-treatment. Data are reported as means ± SEM. ANOVA, *P* < 0.0001; Newman–Keuls multiple comparison post hoc test, *P < 0.05 *vs.* negative (-) Ctrl.

*Allium ursinum* extracts (31.25–1000 µg mL^−1^) were also tested on the mouse myoblast C2C12 cell line under either basal conditions or hydrogen peroxide-induced oxidative stress. Under basal conditions, all extracts were biocompatible, showing only a slight reduction in cell viability at the highest concentrations of the water extract, and never exceeding a 30% decrease in viability that corresponds to the toxicity threshold (Fig. 8A–C). When cells were challenged with hydrogen peroxide (H_2_O_2_, 750 µM), the extracts were partially effective in counteracting the H_2_O_2_-induced reduction in cell viability (Fig. 9A–C). This effect is consistent with the observed scavenging and reducing activities, as well as with the phenolic content of the extracts. Notably, the hydroalcoholic extract (50%, v/v) exerted the strongest protective effect, further suggesting that the biological properties of *A. ursinum* extracts depend not only on phenolic constituents but also on other specialized metabolites.^4^

### 3.4. LC-HRMS/MS dereplication and molecular network of the hydroalcoholic (50%, v/v) leaf extract

Considering the results from biological and eco-toxicological screenings, the hydroalcoholic (50%, v/v) leaf extract was further characterized from both a metabolomic and a pharmacological perspective, as detailed below. The chromatogram is provided in the SI.

**Fig. 10:**
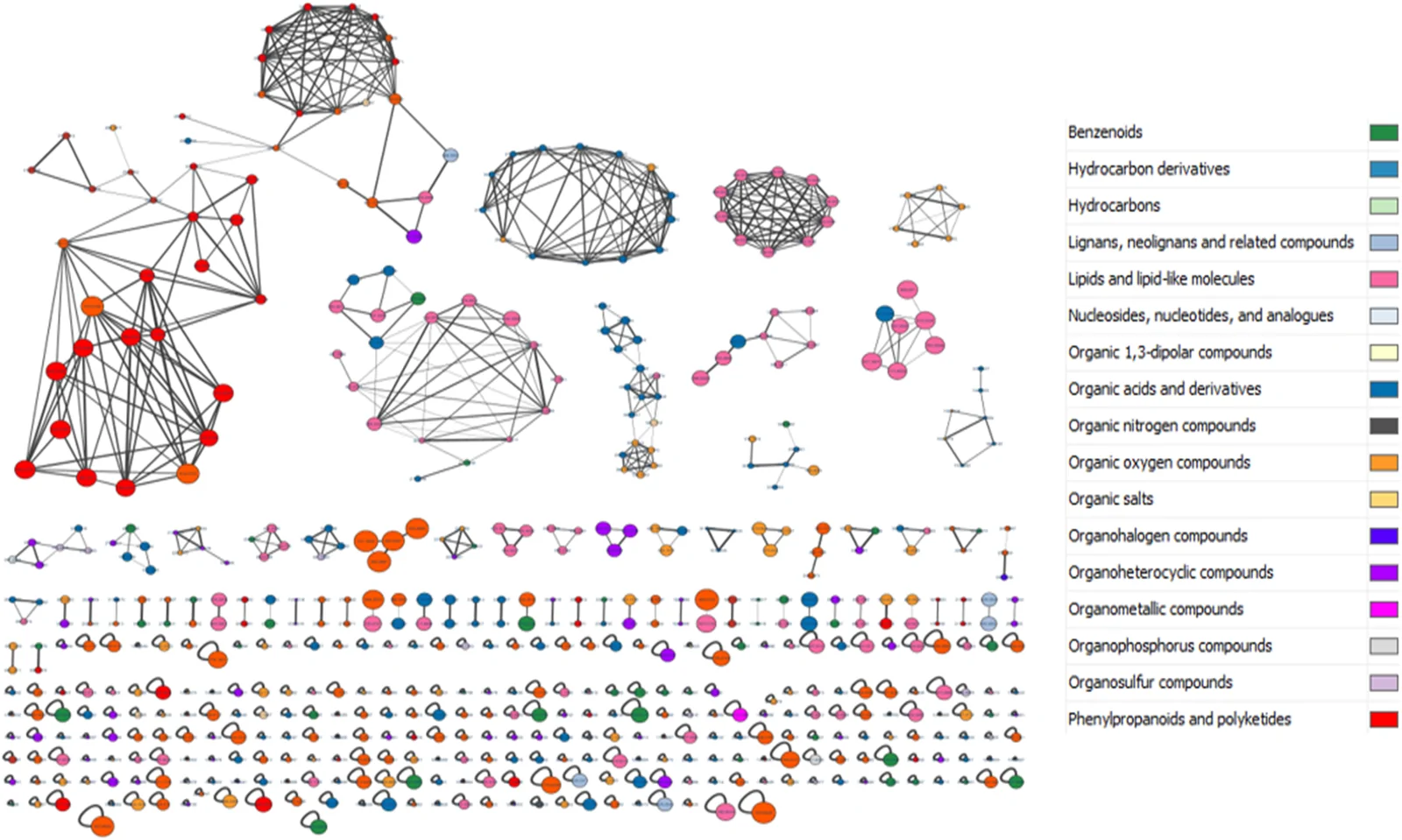
Molecular Network from the dereplication of *A. ursinum*'s Leaves' hydroalcoholic extract.

The comparison with the databases showed 22 different compounds with significant identification match (>0.699) in the hydroalcoholic extract obtained from the *A. ursinum* leaves. The compounds matched from GNPS and Sirius databases were also used to build the metabolomic network of the extract, representated below (Fig. 10). The most predominant classes of specialized metabolites are the phenylpropanoids and the organic acids. However, compounds belonging to the classes of benzenoids, flavonoids, organic oxygenates, organoaminos, organoheterocyclics, phenols, terpenoids, and lignans, neolignans, and related compounds were also identified. From the 22 detected compounds, 15 were identified in the positive mode (Table 8) and 7 under negative ionization (Table 9). The substances alfrutamide (*m*/*z* [M − H]^−^ = 312.1240), kaempferol-3-*O*-rutinoside (*m*/*z* [M − H]^−^ = 593.1514), and kaempferol 3-gentiobioside were the only ones simultaneously detected under both ionization conditions (Fig. S1).

**Table 8:** Identified Molecules in *A. ursinum*'s Leaves' hydroalcoholic extract by GNPS and Sirius with Positive Ionization in Orbitrap

RT	Compound	GNPS cossine index	SIRIUS score	Molecular formule	[M + H]^+^ th.	[M + H]^+^ Exp.	Δ (ppm)	Reported by
0.90	Methylthioadenosine sulfoxide	0.87	0.874	C_11_H_15_N_5_O_4_S	314.09175	314.09160	−0.47757	Barbu *et al.*, 2023
2.19	Kaempferol-3-*O*-glucoside	0.96	—	C_21_H_20_O_11_	449.10784	449.10779	−0.11133	Pârvu *et al.*, 2011
2.20	*S*-(Allylthio)-cysteine	—	0.830	C_6_H_11_NO_2_S_2_	757.21857	757.21832	−0.33016	Barbu *et al.*, 2023
2.36	Kaempferol 3-gentiobioside	0.94	0.814	C_27_H_30_O_16_	611.16066	611.16051	−0.24543	Pârvu *et al.*, 2011
2.50	*S*-allylmercaptoglutathione	—	0.971	C_13_H_21_N_3_O_6_S_2_	380.09445	380.09419	−0.68404	Barbu *et al.*, 2023
2.71	Allithiamine	—	0.824	C_15_H_22_N_4_O_2_S_2_	355.12569	355.12600	0.87293	Barbu *et al.*, 2023
2.74	Kaempferol-3-*O*-galactoside	—	0.780	C_21_H_20_O_11_	449.10784	449.10799	0.33400	Pârvu *et al.*, 2011
2.78	Kaempferol 3-*O*-rutinoside	0.90	0.778	C_27_H_30_O_15_	595.16575	595.16644	1.15934	Pârvu *et al.*, 2011
2.79	Kaempferol	0.73	0.779	C_15_H_10_O_6_	287.05501	287.05514	0.45287	Pârvu *et al.*, 2011
3.33	Ficusin	—	0.733	C_11_H_6_O_3_	187.03897	187.03909	0.64158	Nanusha *et al.*, 2020
3.46	Alfrutamide	0.98	0.830	C_18_H_19_NO_4_	314.13869	314.13859	−0.31833	Acharya Balkrishna, 2023
4.20	Bergapten	0.95	0.840	C_12_H_8_O_4_	217.04954	217.05000	2.11933	Nanusha *et al.*, 2020
4.24	Methoxsalen	0.85	0.718	C_12_H_8_O_4_	217.04954	217.04960	0.27643	Nanusha *et al.*, 2020
4.45	Isopimpinellin	—	0.820	C_13_H_10_O_5_	247.0601	247.06015	0.20238	Nanusha *et al.*, 2020
4.61	Sakuranetin	0.95	0.867	C_16_H_14_O_5_	353.26864	353.26858	−0.16984	Acharya Balkrishna, 2023

**Table 9:** Identified Molecules in *A. ursinum*'s Leaves' hydroalcoholic extract by GNPS in Negative Ionization in Orbitrap

RT	Compound	GNPS cossine index	Molecular formule	[M + H]^+^ th.	[M + H]^+^ Exp.	Δ (ppm)	Reported by
2.28	Salidroside	0.95	C_14_H_20_O_7_	299.11253	299.11366	3.77784	Barbu *et al.*, 2023
2.71	Kaempferol 3-gentiobioside	0.76	C_27_H_30_O_16_	609.14501	609.14557	0.91932	Pârvu *et al.*, 2011
2.78	Kaempferol 3-*O*-rutinoside	0.82	C_27_H_30_O_15_	593.1501	593.15092	1.38245	Pârvu *et al.*, 2011
2.83	Alfrutamide	0.86	C_18_H_19_NO_4_	312.12303	312.12412	3.49221	Acharya Balkrishna, 2023
3.30	Harpagoside	0.91	C_24_H_30_O_11_	493.17044	493.17241	3.99456	Rhetso *et al.*, 2020
3.71	3-*O*-methylquercetin	0.82	C_16_H_12_O_7_	315.04993	315.05099	3.36455	Pârvu *et al.*, 2011
5.96	Cannabidiolic acid	0.73	C_22_H_30_O_4_	357.20604	357.20678	2.07163	Rhetso *et al.*, 2020

The superclass cluster of the phenylpropanoids contains the previous mentioned phenylpropenoic acid amide compound alfrutamide. While no report of this molecule was found about the *A. ursinum* itself, a huge diversity of phenolic compounds are always cited.^26,30,31^ Many articles about the *Allium* genus, however, highlights the alfrutamide presence and its biological effects, particularly on cyclooxygenases and lipoxygenases.^32,33^ Within the phenylpropanoids, three furanocoumarins were observed: ficusin, methoxsalen, and bergapten. The bergapten were already found as a residue from the *A. ursinum* in Germany. These furanocoumarins are reported in the literature with antitumor activity in a variety of cell types and with photosensitizers properties that can cause damage in the skin if followed by sunlight exposure.^34^ Flavonoids, especially quercetins and kaempferols, are also observed in the extracts of several species of the genus, being regularly highlighted by their diverse bioactivities (anti-obesity, anti-cancer, anti-oxidant, and antimicrobial).^32,33^ Four 3-*O*-glycolized derived from the kaempferol were observed in the present study, being already reported in ethanolic extracts from the leaves of *A. ursinum*.^35,36^ While terpenoids, such as cannabidiolic acid and harpagoside, appear more prominently as constituents of essential oils, Rhetso and colleagues^37^ dicated a high terpene content in the leaves of *A. chinense*. Pontin *et al.*^38^ identified several different terpenes in *A. sativum*, as well. These findings demonstrate that terpenoids represent not only a quantitative fraction of the plant metabolome, but also a functionally integral component of its metabolic architecture and physiology, underpinning key aspects of growth, defense, and environmental responsiveness. As for the chemical markers of the species, it is well established in the literature that the leaves of *Allium* species are rich in phenols, flavonoids, and thiosulfinates.^30^ Metabolites belonging to these classes were confirmed through the dereplication workflow. While the phenols and flavonoids identified were consistent with what is reported in the literature, among the classical sulfur markers of the *Allium* genus only *S*-allylcysteine could be identified with sufficient precision. Other characteristic sulfur compounds, such as allicin, alliin, diallyl polysulfides, and vinyldithiins, could not be unequivocally characterized under the applied analytical conditions. Previous studies have shown that ethanolic extracts are particularly rich in polyphenols and flavonoids compared with methanolic, acetonic, or aqueous extracts,^30^ which may help explain the absence of these sulfur compounds at quantifiable or structurally assignable levels in the analyzed samples. Overall, the molecular interrelationships observed highlight the complex biochemical connectivity among plant secondary metabolites and the inherent challenges in achieving a complete chemical characterization of complex matrices such as plant extracts. Even compounds that are structurally or biosynthetically related may display distinct ionization behaviors, fragmentation patterns, or other analytical responses that hinder their identification. Nevertheless, the present work clearly demonstrates the chemical richness and potential of the studied extract.

### 3.5. Protective effects of the 50% (v/v) hydroalcoholic leaf extract in isolated colon exposed to LPS

**Fig. 11:**
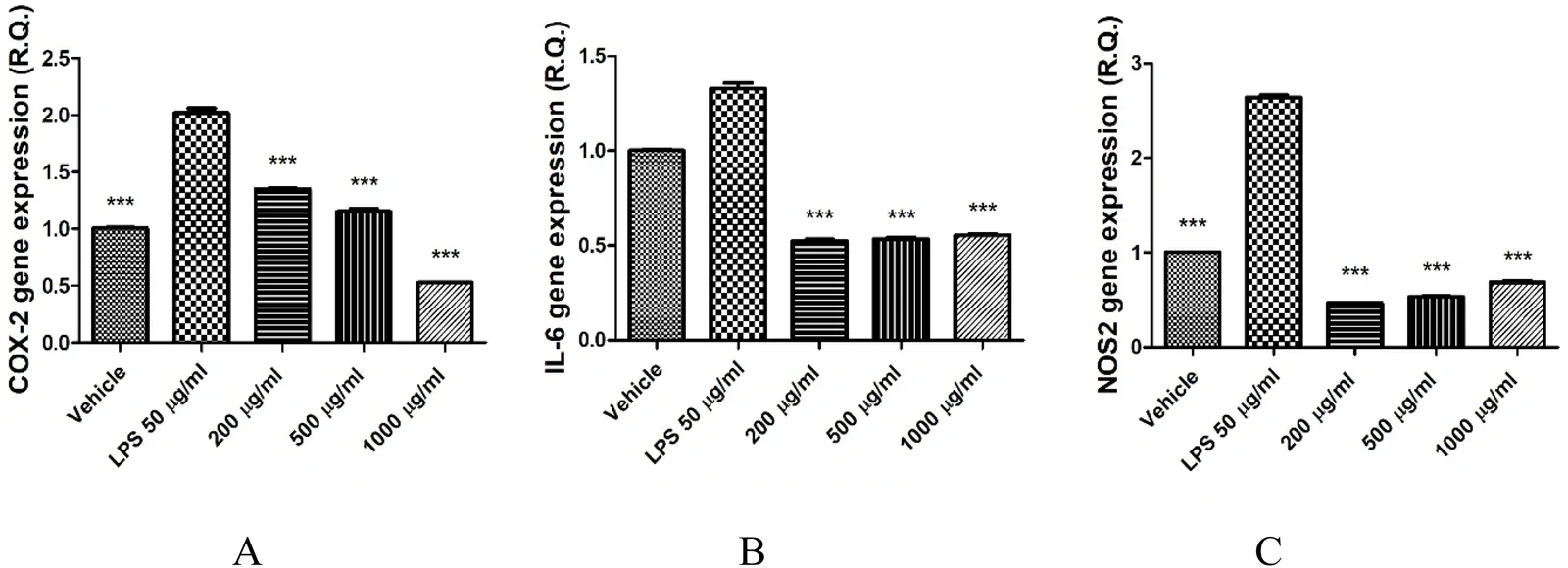
Inhibitory effects induced by hydroalcoholic (50%, v/v) extract (200–1000 µg mL^−1^) from *Allium ursinum* leaves on cyclooxygenase (COX-2) (A), interleukin-6 (IL-6) (B), and inducible nitric oxide synthase (NOS2) (C) gene expression, in isolated mouse colon exposed to *Escherichia coli* lipopolysaccharide (LPS) (50 µg mL^−1^). ANOVA, *P* < 0.0001; Newman–Keuls multiple comparison post hoc test, ***P < 0.001 *vs.* Vehicle.

Based on its observed biocompatibility and protective activity against hydrogen peroxide-induced reduction in C2C12 cell viability, the hydroalcoholic leaf extract (50%, v/v) was subsequently evaluated at the same concentration range (200–1000 µg mL^−1^) in isolated mouse colon tissue exposed to LPS (50 µg mL^−1^). This *ex vivo* model was selected for its feasibility in evaluating the antioxidant and anti-inflammatory properties of plant extracts under conditions of acute LPS-induced toxicity.^13,39^ The extract markedly inhibited the LPS-induced upregulation of COX-2, iNOS, also known as NOS2, and IL-6 gene expression (Fig. 11A–C), thereby supporting the rationale for using phytotherapeutic preparations from *A. ursinum* in the management of clinical symptoms associated with inflammatory diseases of the colon. These anti-inflammatory effects were observed at all tested concentrations, although the concentration-dependent response was observed only in the case of COX-2. This efficacy is likely attributable not only to specific interactions between the extract's phytochemical constituents and the target biomarkers, but also to its scavenging and reducing activities (Table 5). Indeed, hydroxycinnamic acids, flavonoids, and sulfur compounds, among which allicin, have been reported to inhibit the gene expression and plasma levels of these pro-inflammatory factors in both *in vitro* studies and animal models of colitis.^40–43^ However, we cannot rule out the possibility that other phytocompounds may contribute, at least in part, to the antioxidant and anti-inflammatory activities observed in this extract. To further elucidate the molecular mechanisms underlying these effects, a comprehensive *in silico* investigation was conducted, integrating network pharmacology and molecular docking analyses.

### 3.6. *In silico* study

#### 3.6.1. Identification of overlapping targets

**Fig. 12:**
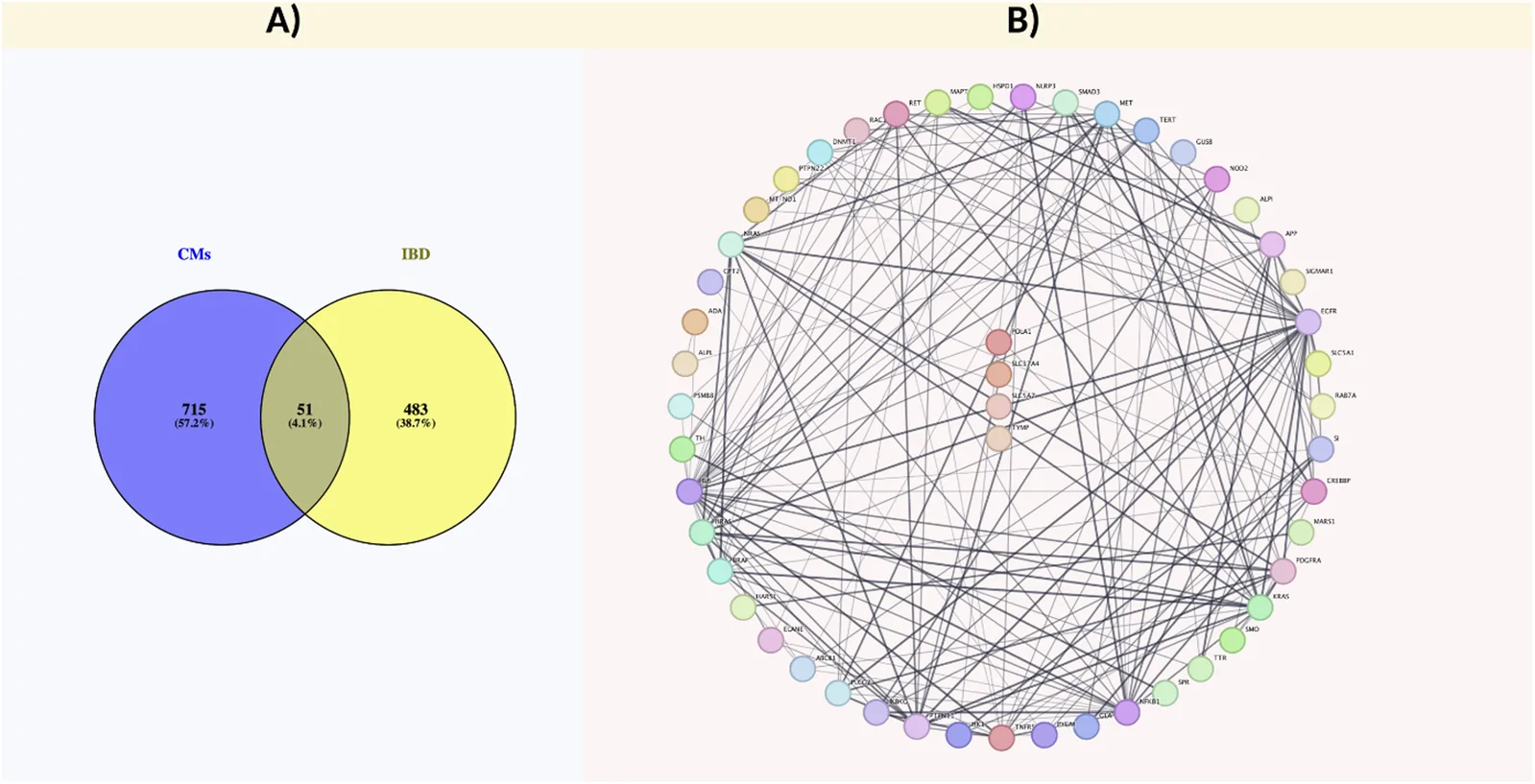
Identification and interaction analysis of overlapping targets related to IBD. (A) Venn diagram showing the overlap between CMs targets and IBD-related genes. (B) PPI network of the 51 overlapping targets.

The predicted targets of the identified compounds and the disease-associated targets were initially organized and visualized as separate networks in Cytoscape. The compound-related target network contained 766 nodes and 2445 edges, while the disease-related target network included 534 nodes and 6790 edges (Fig. S2 and S3). Following target validation and duplicate removal, intersection analysis was carried out to determine the shared genes between the two datasets. This analysis identified 51 overlapping targets (Fig. 12), which were considered candidate genes potentially mediating the pharmacological effects of the identified compounds in the disease context. To investigate the interaction profile of these common targets, a PPI network was generated using the STRING database. The resulting network consisted of 51 nodes and 180 edges, suggesting that the overlapping proteins formed a functionally connected interaction module.

#### 3.6.2. GO and KEGG analysis

**Fig. 13:**
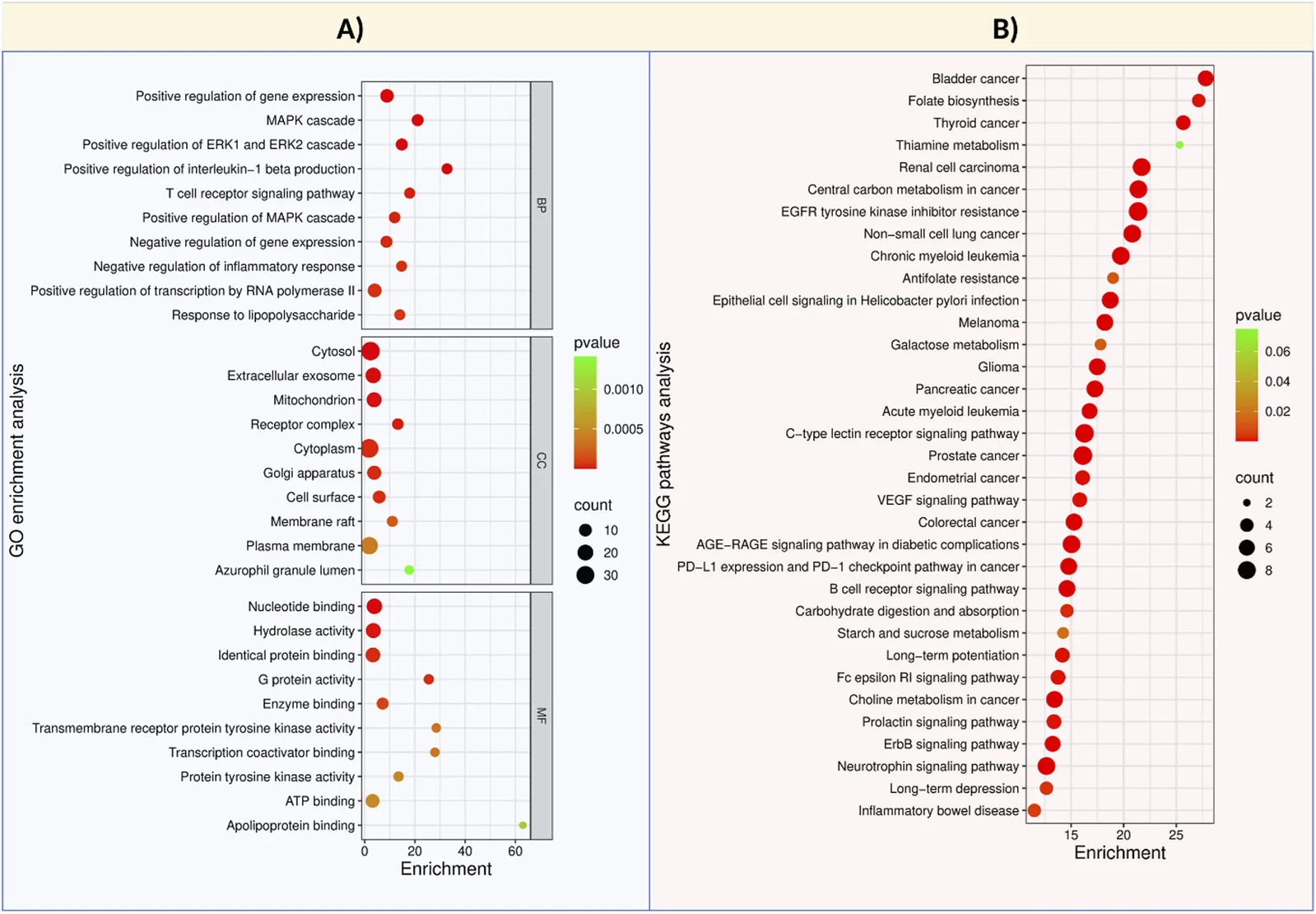
GO (A) and KEGG (B) enrichment analysis of overlapping targets associated with IBD.

GO and KEGG enrichment analyses were performed to investigate the potential biological functions of the overlapping targets. A total of 175 significantly enriched GO terms (*P* < 0.05) were identified, including 107 biological process (BP) terms, 32 cellular component (CC) terms, and 36 molecular function (MF) terms. In the BP category, the overlapping targets were mainly enriched in positive regulation of gene expression, MAPK cascade, positive regulation of ERK1 and ERK2 cascade, positive regulation of interleukin-1 beta production, T cell receptor signaling pathway, positive regulation of MAPK cascade, negative regulation of gene expression, negative regulation of inflammatory response, positive regulation of transcription by RNA polymerase II, and response to lipopolysaccharide. In the CC category, the enriched terms were primarily associated with cytosol, extracellular exosome, mitochondrion, receptor complex, cytoplasm, Golgi apparatus, cell surface, membrane raft, plasma membrane, and azurophil granule lumen. For MF, the targets were mainly involved in nucleotide binding, hydrolase activity, identical protein binding, G protein activity, enzyme binding, transmembrane receptor protein tyrosine kinase activity, transcription coactivator binding, protein tyrosine kinase activity, ATP binding, and apolipoprotein binding (Fig. 13A). KEGG pathway analysis identified 119 significantly enriched pathways (*P* < 0.05). When ranked according to fold enrichment, the most prominent pathways included bladder cancer, folate biosynthesis, thyroid cancer, renal cell carcinoma, central carbon metabolism in cancer, EGFR tyrosine kinase inhibitor resistance, non-small cell lung cancer, chronic myeloid leukemia, C-type lectin receptor signaling pathway, and VEGF signaling pathway. Notably, the inflammatory bowel disease (IBD) pathway was also enriched and included the overlapping targets IL6, SMAD3, NOD2, and NFKB1, supporting the relevance of the identified targets to the disease context (Fig. 13B).

#### 3.6.3. Compound–target network analysis

**Fig. 14:**
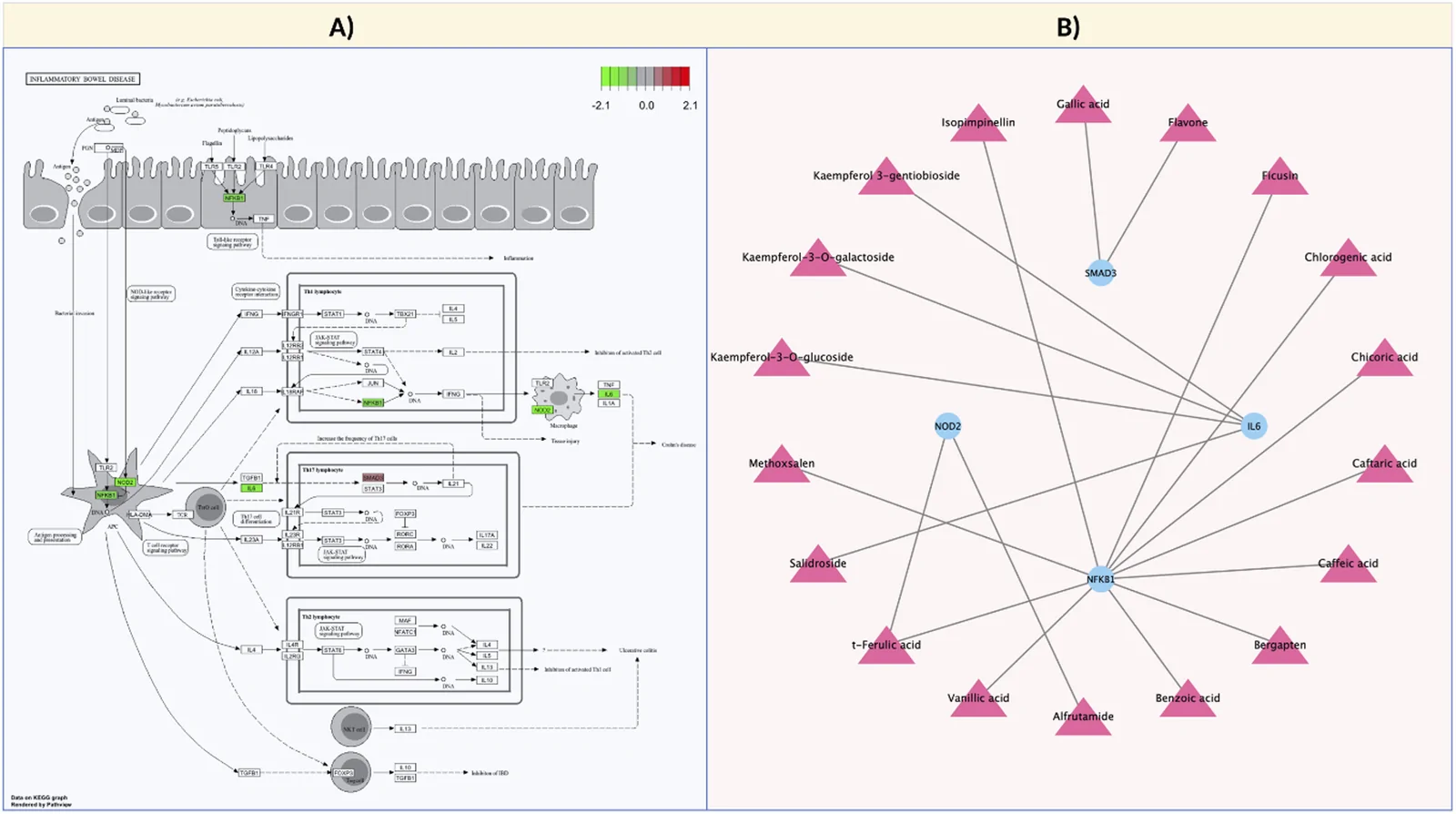
Pathway mapping and compound–target network analysis of the enriched IBD pathway. (A) Pathview mapping of the overlapping targets within the IBD. (B) Compound–target network of the pathway-associated overlapping genes and their corresponding ligands.

To further clarify the interactions between the identified compounds and the key IBD-related targets represented in the enriched IBD pathway, a focused compound–target network was constructed using the pathway-associated overlapping genes and their corresponding ligands (Fig. 14A). The resulting network consisted of 22 nodes and 20 edges, comprising 18 compounds and 4 targets (IL6, NFKB1, NOD2, and SMAD3), and revealed a characteristic multi-compound–multi-target interaction pattern. Among the target nodes, NFKB1 showed the highest degree of connectivity, being linked to 12 compounds, namely alfrutamide, benzoic acid, bergapten, caffeic acid, caftaric acid, chicoric acid, chlorogenic acid, ficusin, isopimpinellin, methoxsalen, *t*-ferulic acid, and vanillic acid, indicating that this target may represent the principal regulatory axis within the network. IL6 displayed the second-highest connectivity and was associated with four compounds, including kaempferol 3-gentiobioside, kaempferol-3-*O*-galactoside, kaempferol-3-*O*-glucoside, and salidroside. In contrast, NOD2 and SMAD3 showed more selective interaction profiles, each being connected to two compounds. NOD2 was linked to alfrutamide and *t*-ferulic acid, whereas SMAD3 was associated with flavone and gallic acid. Notably, alfrutamide and *t*-ferulic acid interacted with both NFKB1 and NOD2, suggesting comparatively broader target coverage than the remaining compounds (Fig. 14B). Overall, the compound–target network suggests that the identified metabolites may modulate the IBD-related pathway through coordinated regulation of inflammatory and immune-associated mediators, with NFKB1 emerging as the most prominent hub-like target. These findings further support the multi-component and multi-target pharmacological profile proposed by the network pharmacology framework.

#### 3.6.4. Binding propensity and protein-ligand interaction

**Fig. 15:**
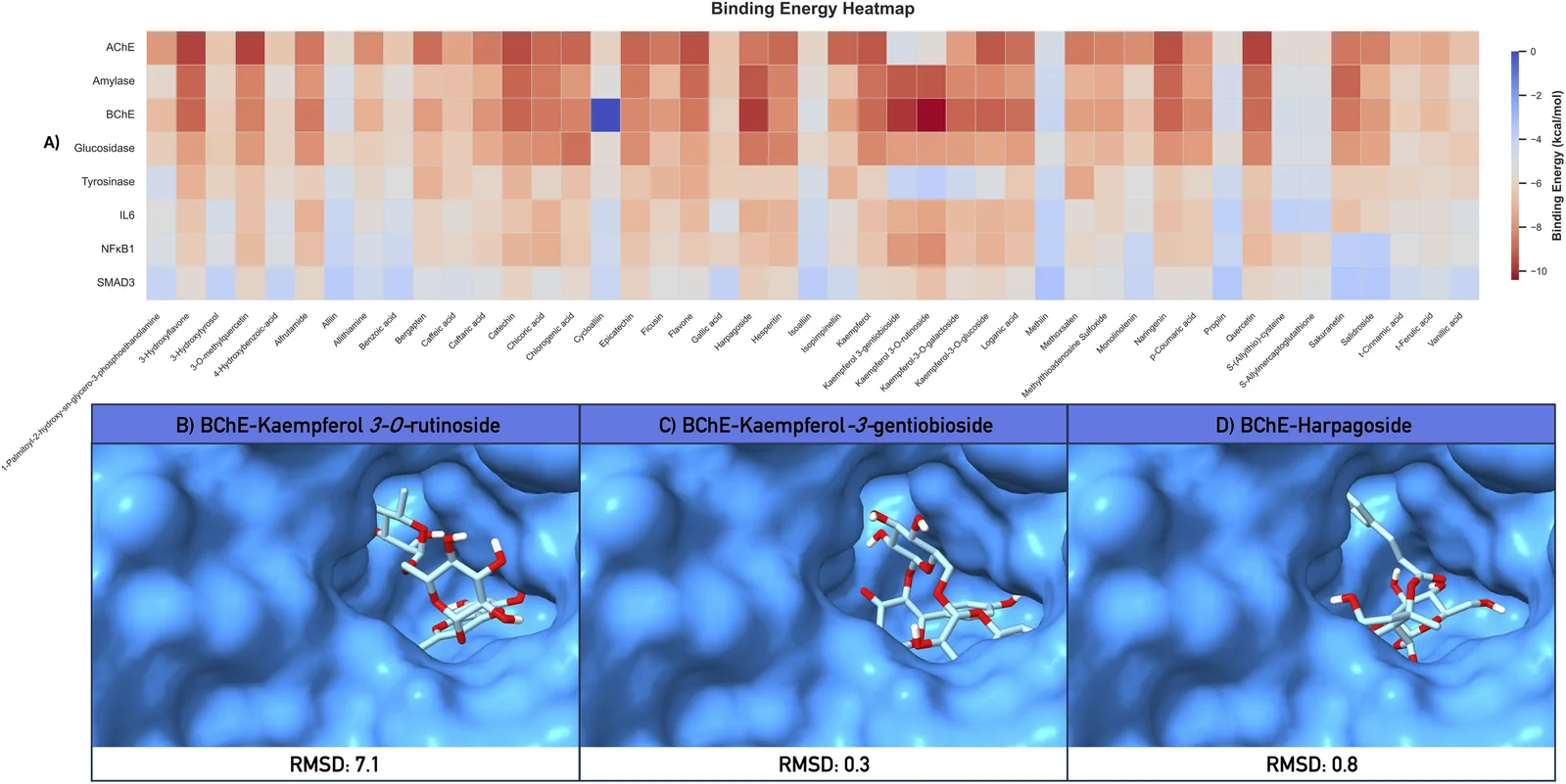
(A) Heatmap of docking (binding energy) scores and protein–ligand interaction maps of selected complexes: (B) BChE-kaempferol 3-*O*-rutinoside; (C) BChE-kaempferol 3-*O*-gentiobioside; (D) BChE-harpagoside.

The docking analysis across multiple targets, including AChE, BChE, amylase, glucosidase, tyrosinase, IL-6, NF-κB, and SMAD3, revealed that the key compounds kaempferol-3-*O*-rutinoside, kaempferol 3-gentiobioside, and harpagoside, consistently exhibited strong binding affinities, particularly toward BChE (−9 to −11 kcal mol^−1^) (Fig. 15A–C).

For BChE, kaempferol 3-*O*-rutinoside formed hydrogen bonds with Glu197 and Ser198 but exhibited comparatively weaker interaction persistence (Fig. 15B) and kaempferol 3-gentiobioside demonstrated the most favorable interaction profile, forming multiple hydrogen bonds with catalytic residues such as Ser198, His438, and Glu325, along with π–π stacking interactions with Trp82 and Phe329, ensuring strong stabilization within the active site gorge (Fig. 15B). The combination of these interactions supports strong ligand accommodation within the enzyme cavity and reflects the ability of bulky natural products to effectively bind BChE.^44^

Harpagoside also showed robust binding through hydrogen bonding with Ser198 and Tyr332 and hydrophobic contacts with Trp82 (Fig. 15D). Against AChE, these compounds displayed moderate-to-strong binding (−7 to −9 kcal mol^−1^), stabilized by hydrogen bonds with residues such as Ser203 and His447 and π–π interactions with Trp86 and Tyr337, indicating dual cholinesterase inhibition potential.

For amylase and glucosidase, binding affinities were slightly lower (−6 to −8 kcal mol^−1^), with interactions primarily driven by hydrogen bonding with catalytic residues (Asp197, Glu233 in amylase; Asp518, Glu521 in glucosidase) and van der Waals contacts within the active pocket. In the case of tyrosinase, moderate binding involved coordination-like interactions with active-site residues (His85, His263) and hydrogen bonding networks.

These compounds also showed appreciable binding toward inflammatory and signaling proteins such as IL-6, NF-κB, and SMAD3 (−7 to −10 kcal mol^−1^), where stabilization was mediated through hydrogen bonds with polar residues and hydrophobic interactions within regulatory domains, suggesting potential anti-inflammatory activity. The binding profiles indicate that hydrogen bonding, π–π stacking, and hydrophobic interactions collectively govern ligand affinity across targets, with kaempferol 3-gentiobioside consistently demonstrating the most optimal and stable interaction network.

## 4 Conclusion

Concluding, the present study investigated, through a multilevel approach, the potential health-promoting properties of *Allium ursinum* extracts from leaves that represent a traditional food, among Apennine populations. The extracts were prepared using polar solvents like water and hydroalcoholic solutions, in order to mimic traditional home-made preparations. The extracts exhibited a complex metabolomic profile largely characterized by sulfur-containing compounds, with methiin and alliin as the predominant constituents. The water extract was particularly rich in sugars, amino acids, and organic acids, while hydroalcoholic extracts selectively concentrated polyphenolic compounds. These latter extracts demonstrated the highest antioxidant capacity and enzyme inhibitory effects, which were positively associated with their total phenolic and flavonoid levels. Notably, the hydroalcoholic (50%, v/v) leaf extract markedly reduced LPS-induced expression of IL-6, COX-2, and iNOS in isolated colon tissue *ex vivo*, indicating significant anti-inflammatory activity. The same extract revealed to be rich in flavonoids. Particularly, kaempferol derivatives showed appreciable putative affinities towards the tested enzymes and inflammatory and signaling proteins, among which IL-6. Therefore, the inhibitory effect induced by the extract on colon IL-6 gene expression is corroborated, at least in part, by computational approaches. Taken together, this multilevel evidence supports the biofunctional relevance of *A. ursinum* leaf extracts and lays a solid foundation for future studies aimed at validating their efficacy and mechanisms of action using complementary analytical and pharmacological approaches. Finally, the findings of this study may provide additional insight into the importance of enhancing knowledge and valorization of *A. ursinum*, a precious botanical, nutritional, particularly phytoalimurgic, and medicinal resource. Although its use was traditionally widespread among Apennine populations, renewed scientific attention to this species may foster a more comprehensive understanding of the role of territorial biodiversity and its positive, decisive impact on human health. In this regard, the present work aligns with a One Health perspective, emphasizing the recovery of traditional plant-based knowledge embedded in the natural environments where it originated and has been preserved for centuries.

## Ethical statement

Housing conditions and experimentation procedures were strictly in agreement with the European Community ethical regulations (EU Directive no. 63/2010) on the care of animals for scientific research. According to the recognized principles of “Replacement, Refinement and Reduction in Animals in Research”, colon specimens were obtained as residual material from vehicle-treated animals randomized in our previous experiments, approved by the local ethical committee (‘G. d’Annunzio’ University, Chieti, Italy) and Italian Health Ministry (Project no. F4738.N.5QP).

## Author contributions

Alessandra Acquaviva: methodology, formal analysis, investigation. Simonetta Cristina Di Simone: methodology, formal analysis, investigation, writing – original draft. Raimundo Gonçalves de Oliveira-Júnior: methodology, formal analysis, investigation, writing – original draft. Leandro Machado Rocha: methodology, formal analysis. Thomas Gaslonde: methodology, formal analysis, investigation. Leonardo de Assunção Pinto: methodology, formal analysis, investigation. Mattia Spano: methodology, formal analysis, investigation. Mehmet Veysi Cetiz: methodology, formal analysis, investigation. Abdullahi Ibrahim Uba: methodology, formal analysis, investigation. Annalisa Chiavaroli: methodology, formal analysis, investigation. Lucia Recinella: methodology, formal analysis, investigation. Sheila Leone: methodology, formal analysis, investigation. Gokhan Zengin: methodology, formal analysis, investigation. Fatma Tunali: investigation. Mariachiara Gabriele: methodology, formal analysis, investigation. Luigi Brunetti: methodology, formal analysis, investigation. Giustino Orlando: methodology, formal analysis, investigation, Luigi Menghini: methodology, formal analysis, investigation, writing – original draft, funding, resources. Claudio Ferrante: methodology, formal analysis, investigation, coordination of the study, writing – original draft, funding, resources. All authors have read and agreed to the published version of the manuscript.

## Conflicts of interest

The authors declare that the research was conducted in the absence of any commercial or financial relationships that could be construed as a potential conflict of interest.

## Abbreviations

ABTS: 2,2′-Azinobis-(3-ethylbenzothiazoline-6-sulfonic acid)
AChE: Acetylcholinesterase
AuH50: Hydroalcoholic (50%, v/v) extract from *Allium ursinum* leaves
AuH70: Hydroalcoholic (70%, v/v) extract from *Allium ursinum* leaves
AuW: Water extract from *Allium ursinum* leaves
BChE: Butyrylcholinesterase
COX-2: Cyclooxygenase-2
CUPRAC: CUPric reducing antioxidant capacity
DPPH: 1,1-Diphenyl-2-picrylhydrazil
FRAP: Ferric reducing antioxidant power
GAE: Gallic acid equivalents
IL-6: Interleukin-6
iNOS: Inducible nitric oxide synthase
LPS: Lipopolysaccharide
MD: Molecular dynamics
MTT: 3-(4,5-Dimethylthiazol-2-yl)-2,5-diphenyltetrazolium bromide
RE: Rutin equivalents
RMSD: Root mean square deviation
RMSF: Root mean square fluctuation
SASA: Solvent accessible surface area
TE: Trolox equivalents
TFC: Total flavonoid content
TPC: Total phenolic content

## Supplementary Material

RA-OLF-D6RA04402F-s001

## Data Availability

Data will be requested from authors. Supplementary information (SI): assays for total phenolics, total flavonoids, antioxidant effect and enzyme inhibition paragraph; allelopathy assay paragraph; cell culture paragraph; *ex vivo* study paragraph; Fig. S1–2 and Table S1. See DOI: https://doi.org/10.1039/d6ra04402f.
