# Supplementary material for: Allium ursinum L. from the apennine flora: a phytoalimurgic resource for modulating gut inflammation
Source: RSC Adv. 2026 Aug 28;16(42):51067–88. doi: 10.1039/d6ra04402f (PMC13523655; doi:10.1039/d6ra04402f)
Supplement: Supplementary file 1 [file RA-016-D6RA04402F-s001.pdf]

Supplementary Information (SI) for RSC Advances.
This journal is © The Royal Society of Chemistry 2026

Title: ***Allium ursinum*** **L. from the Apennine Flora: a phytoalimurgic resource for modulating gut inflammation**

Authors: Simonetta Cristina Di Simone[1**], Mariachiara Gabriele[1**], Alessandra Acquaviva[1], Fatma Tunali[1], Annalisa Chiavaroli[1], Giustino Orlando[1], Luigi Brunetti[1], Lucia Recinella[1], Sheila Leone[1], Leonardo de Assunção Pinto[2,3], Thomas Gaslonde[4], Leandro Machado Rocha[3], Raimundo Gonçalves de Oliveira-Júnior[4], Mattia Spano[5], Mehmet Veysi Cetiz[6], Abdullahi Ibrahim Uba[7], Gokhan Zengin[8], Luigi Menghini[1], Claudio Ferrante[1*]

[1]Botanical Garden "Giardino dei Semplici", Department of Pharmacy, "G. d'Annunzio" Chieti-Pescara, via dei Vestini 31, 66100 Chieti, Italy; simonetta.disimone@unich.it; mariachiara.gabriele@phd.unich.it; alessandra.acquaviva@unich.it; annalisa.chiavaroli@unich.it; giustino.orlando@unich.it; luigi.brunetti@unich.it; lucia.recinella@unich.it; sheila.leone@unich.it; turali.fatma@gmail.com

[2] Post-Graduation Program in Vegetal Biotechnology and Bioprocesses, Rio de Janeiro Federal University, Rio de Janeiro, CEP 21941-590, Brazil; leodeasspinto@gmail.com

[3] Laboratory of Natural Products Technology, Department of Pharmaceutical Technology, Faculty of Pharmacy, Fluminense Federal University, Niterói, CEP 24241-000, Brazil; leandromr@id.uff.br

[4] Cibles Therapeutiques et Conception de Medicaments (CiTCoM), UMR CNRS 8038, Faculty of Pharmacie, Université Paris Cité, 75006, Paris, France; raimundo.goncalves-de-oliveira-junior@u-paris.fr; thomas.gaslonde@u-paris.fr

[5] Department for the Promotion of Human Science and Quality of Life, San Raffaele University, Via di Val Cannuta 247, Rome 00166, Italy; mattia.spano@uniroma5.it

[6]Department of Medical Biochemistry, Faculty of Medicine, Harran University, Sanliurfa, 63290, Turkey, mvcetiz@gmail.com

[7]Department of Biostatistics and Medical Informatics, Faculty of Medicine, Istinye University, 34396 Istanbul, Türkiye, abdullahi.iu2@gmail.com

[8]Department of Biology, Science Faculty, Selcuk University, 42130 Konya, Turkey, gokhanzengin@selcuk.edu.tr

**These authors equally contributed to the study.
*Corresponding Author: Prof. Claudio Ferrante (claudio.ferrante@unich.it)

Assays for Total phenolics, Total flavonoids, Antioxidant effect and Enzyme inhibition
The determination of total phenolic (TPC) and flavonoid (TFC) levels in the extracts was carried out based on previously established protocols (Bibi Sadeer et al., 2020). Standard compounds gallic acid (GA) and rutin (R) were employed for calibration purposes during the assays, and the results were presented as gallic acid equivalents (GAE, mg GAE/g dry extract) and rutin equivalents (RE, mg RE/g dry extract), for TPC and TFC respectively. Antioxidant evaluations were conducted in accordance with established procedures, as delineated in a previous study (Grochowski et al., 2017). The radical scavenging capacities of the extracts were measured using a variety of assays, including ferric reducing antioxidant power (FRAP), CUPric reducing antioxidant capacity (CUPRAC), 1,1-diphenyl-2-picrylhydrazil (DPPH), and 2,2′-azinobis-(3-ethylbenzothiazoline-6-sulfonic acid) (ABTS). The results were expressed as milligrams of Trolox equivalents (TE) per gram of dried extract. The total antioxidant potential was subsequently determined using the phosphomolybdenum (PBD) assay and reported in mmol TE/g. Furthermore, the metal chelation activity (MCA) was quantitatively assessed by measuring the milligrams of EDTA equivalents (EDTAE) per gram of extract. Enzyme inhibitory assays were conducted against acetylcholinesterase (AChE), butyrylcholinesterase (BChE), tyrosinase, amylase, and glucosidase using the tested extracts, according to validated protocols reported in the literature (Grochowski et al., 2017). The inhibitory activities toward AChE and BChE were subsequently quantified in milligrams of galanthamine equivalents (GALAE) per gram of dry extract. The inhibitory potential of amylase and glucosidase was expressed in acarbose equivalents (ACAE), while tyrosinase inhibition was assessed in terms of kojic acid equivalents (KAE) per gram of dry extract.

Allelopathy Assay
A Petri dish-based experiment was carried out to evaluate the potential phytotoxic activity of *A. ursinum* water and hydroalcoholic extracts in a concentration range from 40 to 2.5 mg/mL. Commercial seeds of *Cichorium intybus* L. and *Dicondra repens* were selected for the test due to their fast germination rate and sensitivity. The producers guarantee a germination rate of the seeds >70%, also confirmed by preliminary germination tests. The assay was conducted in 90 mm Petri dishes, each containing a double layered filter paper disk, previously soaked with 3 mL of the plant extract at the different concentrations. Distilled water was used as negative control. The seeds of *C. intybus* and *D. repens* were surface-sterilized using a diluted (NaClO:dH2O, 1:9) commercial bleaching liquid for 10 minutes. After thoroughly rinsing the seeds with sterile distilled water to remove any traces of bleach, 10 seeds of the corresponding seed variety were arranged on the filter paper disks, previously divided into 2 distinct areas. Petri dishes were then sealed with parafilm, to ensure a closed-system model and incubated in darkness at room temperature (25±2°C) for 96h. The criterion for determining germination was the emergence of a radicle at least 2 mm in length, exhibiting typical geotropic curvature. Seeds that only swelled but did not fully germinate were excluded. Radicle length was measured and categorized by elongation rate: low (<0.4 cm), medium (0.5-0.9 cm), or high (>1 cm). The number of germinated seeds and their radicle lengths were recorded after 96 hours, and results were reported as mean Germination Percentage and Growth Percentage, that is the mean of seedlings length compared to control samples. Experiments were conducted in triplicate.

Cell Culture
C2C12 myoblasts, a murine skeletal muscle cell line commonly used as a model for oxidative stress and inflammatory responses, were chosen for this study due to their high sensitivity to pro-oxidant stimuli, such as hydrogen peroxide (H_2O_2). The cells were cultured in DMEM (Dulbecco's Modified Eagle Medium) supplemented with 20% fetal bovine serum (FBS) and 1% penicillin−streptomycin, in a humidified atmosphere of 5% CO_2 at 37 °C. Cells were

routinely monitored under an inverted microscope to ensure proper morphology and confluence. Upon reaching approximately 70% confluence, cells were detached using 0.05% trypsin-EDTA, passaged, and seeded at appropriate densities for subsequent assays.

The MTT assay was used to evaluate the effect of the tested extracts on the viability of C2C12 cells. This assay, widely employed to assess cell viability and cytotoxicity, relies on the reduction of MTT (3-(4,5-dimethylthiazol-2-yl)-2,5-diphenyltetrazolium bromide) by mitochondrial dehydrogenases in viable cells to produce insoluble formazan crystals. The purple formazan was dissolved in DMSO and quantified spectrophotometrically at 570 nm.

To assess cell viability under baseline conditions, C2C12 cells were seeded in 96-well plates at a density of 5×10^3 cells/well and allowed to adhere for 24 h. The extracts were then administered at final concentrations of 200, 500, and 1000 μg/mL for 24 h. After treatment, MTT solution (5 mg/mL) was added to each well and incubated for 3 h at 37 °C. Formazan crystals were solubilized in DMSO, and absorbance was measured at 570 nm using a microplate reader. Control wells containing medium without cells were used as blanks, and cell viability was calculated relative to the untreated control wells.

For evaluating the effect of the extracts under H_2O_2-induced oxidative stress, the same experimental setup was used. Following 24 h treatment with the extracts, H_2O_2 was added at final concentrations of 500 μM for 3 h to induce oxidative stress. After H_2O_2 exposure, the medium was replaced with MTT solution (5 mg/mL) and incubated for 3 h at 37 °C. Formazan crystals were dissolved in DMSO and absorbance was measured at 570 nm. The control wells containing medium without cells were used as blanks, and cell viability was determined relative to the untreated control wells.

Ex Vivo Study

Adult C57/BL6 mice (3-month-old, weight 20-25 g) were housed in Plexiglas cages (2-4 animals per cage; 55 cm × 33 cm × 19 cm) and maintained under standard laboratory conditions (21 ± 2 °C; 55 ± 5% humidity) on a 14/10 h light/dark cycle, with ad libitum access to water and food. Housing conditions and experimentation procedures were strictly in agreement with the European Community ethical regulations (EU Directive no. 63/2010) on the care of animals for scientific research. According to the recognized principles of "Replacement, Refinement and Reduction in Animals in Research", colon specimens were obtained as residual material from vehicle-treated animals randomized in our previous experiments, approved by the local ethical committee ('G. d'Annunzio' University, Chieti, Italy) and Italian Health Ministry (Project no. F4738.N.5QP). The fragments of colon tissue were collected from euthanized mice, were maintained in an humidified incubator with 5% CO_2 at 37°C for 4 h (incubation period), in RPMI buffer with added bacterial LPS (50 μg/mL) and in the presence of water and hydroalcoholic extracts of *A. ursinum* (200-1000 μg/mL). In mouse colon, the gene expression of COX-2, NOS2 and IL-6 was conducted as previously reported (Orlando et al., 2021). Briefly, total RNA was extracted from colon specimens using TRI Reagent (Sigma-Aldrich, St. Louis, MO). Contaminating DNA was be removed using 2 units of RNase-free DNase 1 (DNA-free kit, Ambion, Austin, TX). The RNA concentration was quantified at 260 nm by spectrophotometer reading (BioPhotometer, Eppendorf, Hamburg, Germany) and its purity was assessed by the ratio at 260 and 280 nm readings. The quality of the extracted RNA samples was also determined by electrophoresis through agarose gels and staining with ethidium bromide, under UV light. One microgram of total RNA extracted from each sample in a 20 μL reaction volume was reverse transcribed using a High Capacity cDNA Reverse Transcription Kit (Thermo Fisher Scientific Inc., Monza, Italy). Reactions will be incubated in a 2720 Thermal Cycler (Thermo Fisher Scientific Inc.) initially at 25 °C for 10 min, then at 37 °C for 120 min, and finally at 85 °C for 5 s. Gene expression was determined by quantitative real-time PCR using TaqMan probe-based chemistry. PCR primers and TaqMan probes, including β-

actin used as the housekeeping gene, were purchased from Thermo Fisher Scientific Inc. The real-time PCR was carried out in triplicate for each cDNA sample in relation to each of the investigated genes. Data will be elaborated with the Sequence Detection System (SDS) software version 2.3 (Thermo Fisher Scientific Inc.). Gene expression will be relatively quantified by the comparative $2^{-\Delta\Delta Ct}$ method (Livak and Schmittgen, 2001).

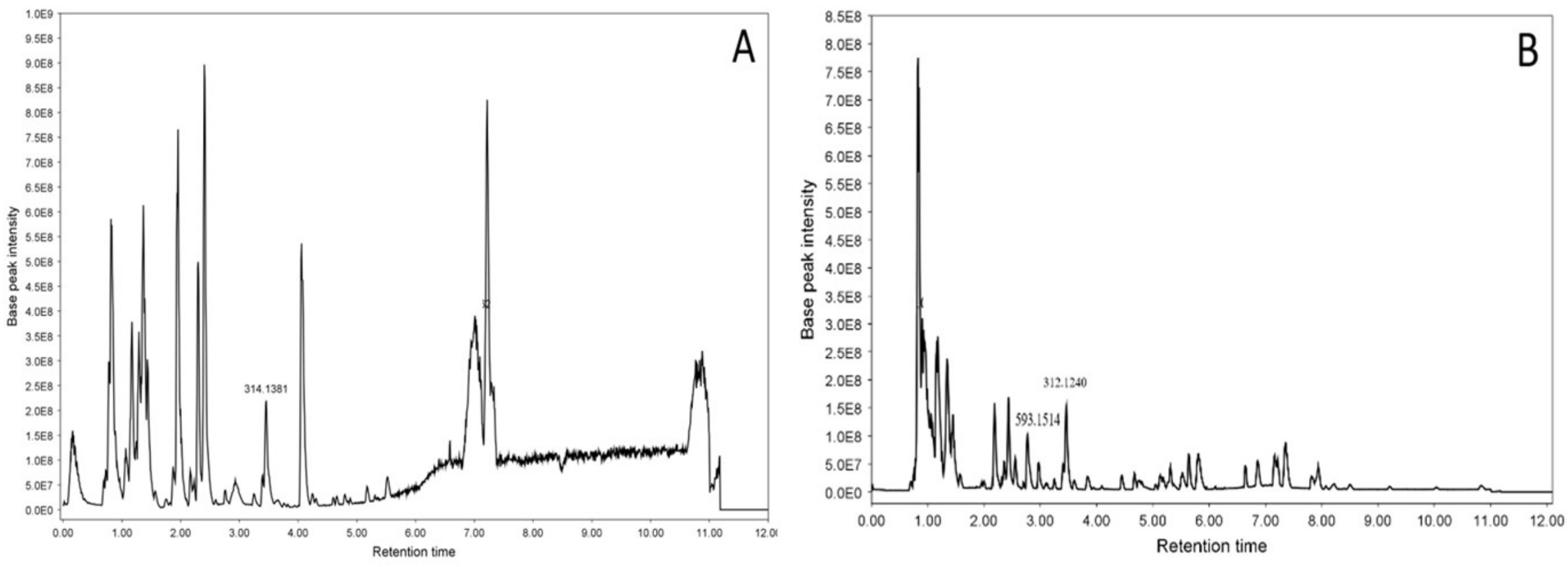

Figure S1. Chromatograms obtained by LC-HRMS/MS in positive (A) and negative (B) ionization modes of *A. ursinum*'s Leaves' hydroalcoholic extract

A. Chromatogram of positive ionization; peak m/z 314.1386, identified as Alfrutamide $[M+H]^+$

B. Chromatogram of negative ionization; peak m/z 593.1514, identified as Kaempferol-3-O-rutinoside $[M-H]^-$; peak m/z 312.1240, identified as Alfrutamide $[M-H]^-$.

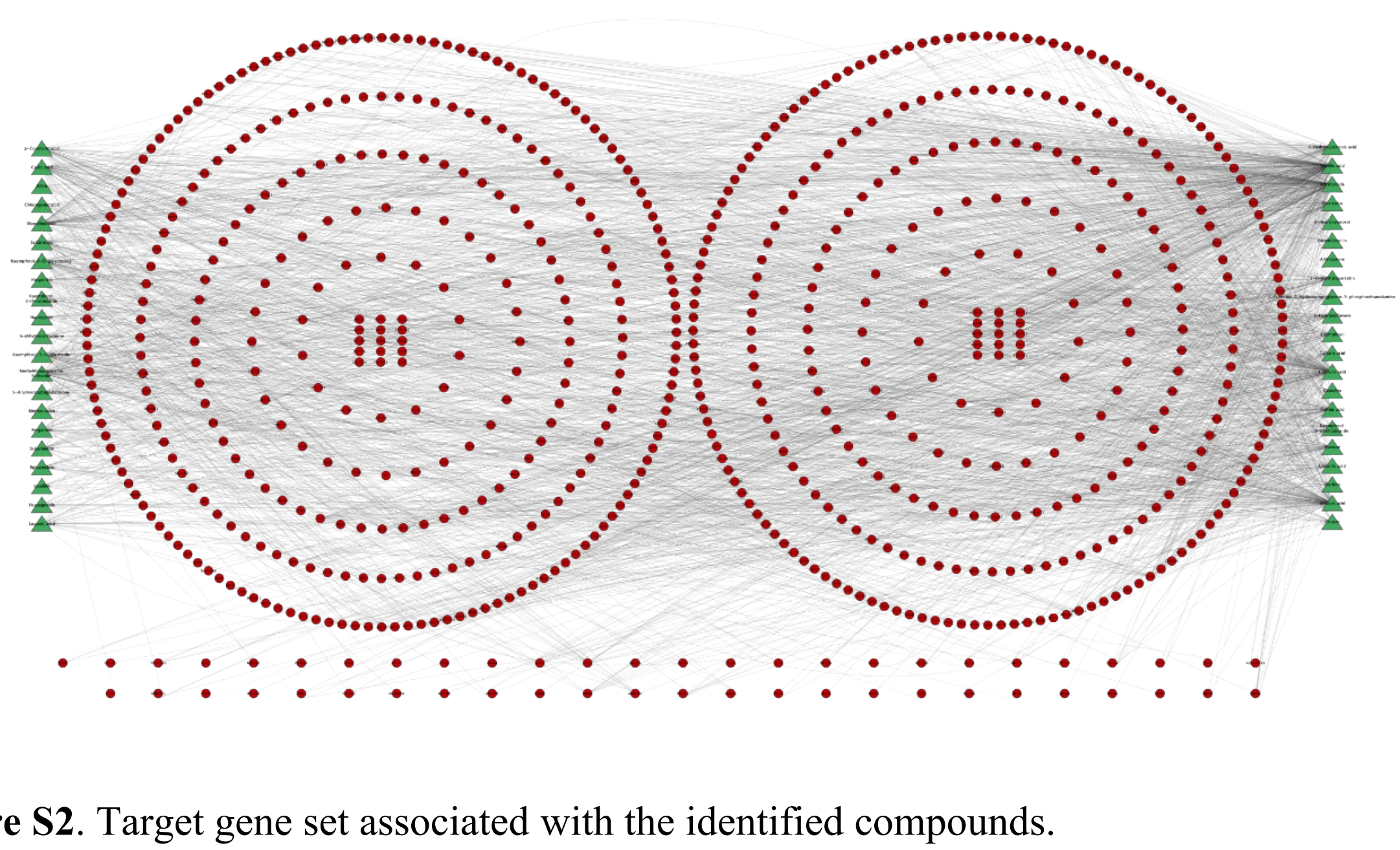

Figure S2. Target gene set associated with the identified compounds.

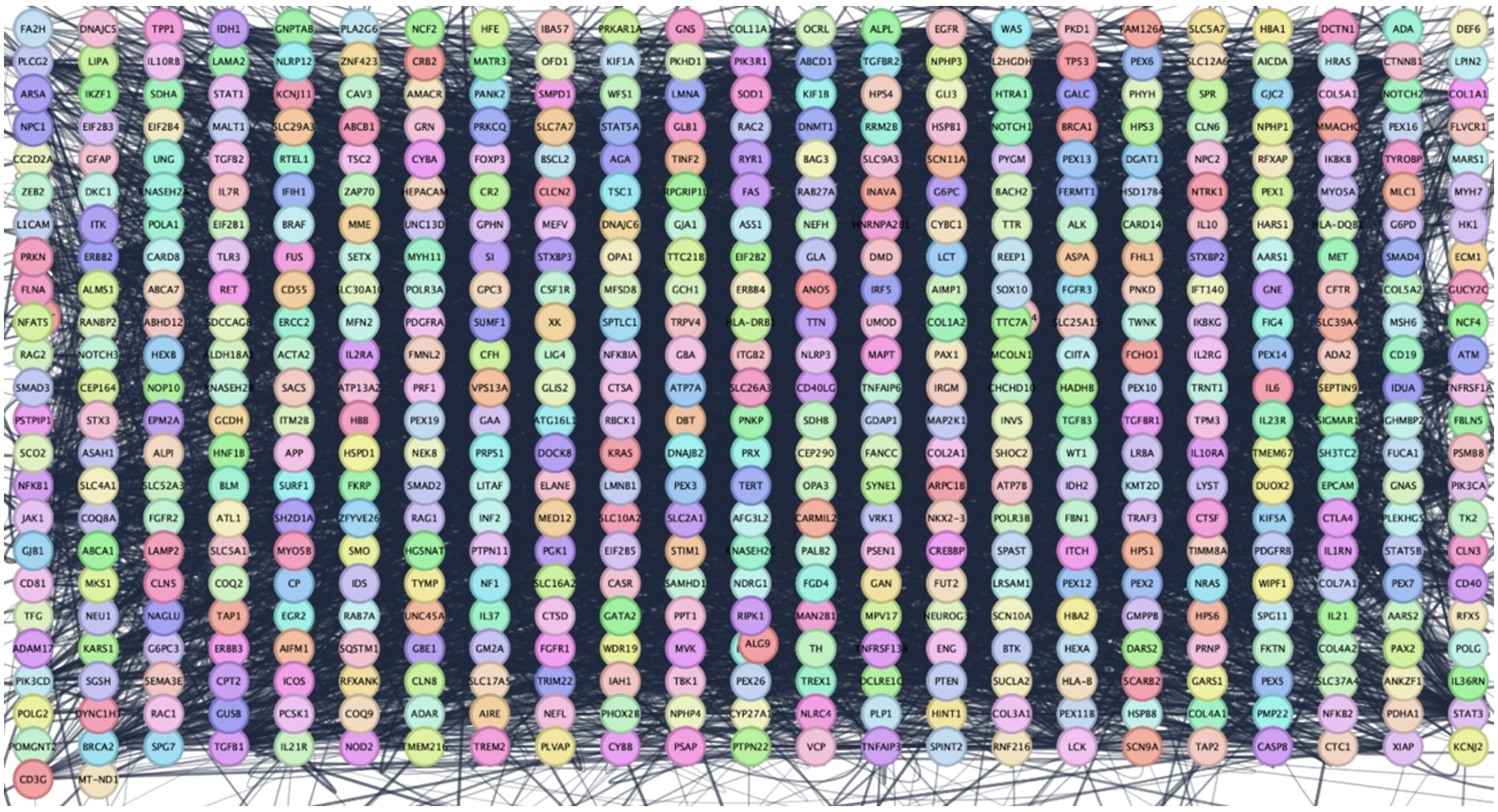

Figure S3. IBD-related gene set obtained from the GeneCards database.

Table S1. Grid center and size used for docking the most abundant phytochemicals in the extracts into the selected enzyme and protein targets.

**Target Protein**	**PDB ID**	**Center X, Y, Z (Å)**	**Grid Size X, Y, Z (Å)**
AChE	7E3H	-54.44, 32.90, -28.65	25, 25, 25
BChE	6EQP	32.16, -16.33, 40.73	25, 25, 25
α-amylase	2QV4	14.188, 48.964, 22.886	28, 28, 24
α-glucosidase	7KBJ	-18.79, 2.18, 15.75	25, 25, 25
Tyrosinase	6QXD	21.81, 12.22, 91.40	25, 25, 25
IL6	5fuc	-56.346, -13.466, 7.413	28, 38, 48
NFκB1	8TQD	13.62, 6.958, -0.612	88, 88, 88
SMAD3	1ozj	32.021, 2.178, 15.837	30, 30 46

References

Grochowski, D.M., Uysal, S., Aktumsek, A., Granica, S., Zengin, G., Ceylan, R., Locatelli, M., Tomczyk, M., 2017. In vitro enzyme inhibitory properties, antioxidant activities, and phytochemical profile of *Potentilla thuringiaca*. Phytochemistry letters 20, 365-372.

Livak, K.J., Schmittgen, T.D., 2001. Analysis of relative gene expression data using real-time quantitative PCR and the 2− ΔΔCT method. methods 25(4), 402-408.

Orlando, G., Chiavaroli, A., Adorisio, S., Delfino, D. V., Brunetti, L., Recinella, L., Leone, S., Zengin, G., Acquaviva, A., Angelini, P., Flores, G. A., Venanzoni, R., Di Simone, S. C., Di Corpo, F., Mocan, A., Menghini, L., & Ferrante, C. (2021). Unravelling the Phytochemical

Composition and the Pharmacological Properties of an Optimized Extract from the Fruit from *Prunus mahaleb* L.: From Traditional Liqueur Market to the Pharmacy Shelf. *Molecules (Basel, Switzerland)*, *26*(15), 4422. https://doi.org/10.3390/molecules26154422
Mustafa, A.M., Angeloni, S., Abouelenein, D., Acquaticci, L., Xiao, J., Sagratini, G., Maggi, F., Vittori, S., Caprioli, G., 2022. A new HPLC-MS/MS method for the simultaneous determination of 36 polyphenols in blueberry, strawberry and their commercial products and determination of antioxidant activity. Food Chemistry 367, 130743.
